# Placentas from healthy women living in a cutaneous leishmaniasis endemic region in Mexico harbor *Leishmania mexicana* parasites: Potential implications for pregnancy and neonatal outcomes

**DOI:** 10.1371/journal.pntd.0014367

**Published:** 2026-09-15

**Authors:** Rodrigo X. Armijos, Brandon A. Berger, Alejandra González Ayala, Miriam A. Delgado-Hernandez, José Luis Acosta-Patiño, Esmelin Trinidad-Vázquez, Liliana Aline Fernández-Urrutia, Elizabeth Baz-Rojas, Javier Mancilla-Galindo, Candelaria Frías Selván, Juana Ortíz-Avalos, Eva Cristina Avalos-Ortiz, Frida Häberle, Miriam Torres-Vasquez, M. Margaret Weigel, Allison H. Bartlett, Yuriria Paredes, Miroslava Avila-García, Magdalena Aguirre García, Norma Galindo-Sevilla

**Affiliations:** 1 Global Environmental Health Laboratory, Department of Environmental & Occupational Health, Indiana University-Bloomington School of Public Health, Bloomington, Indiana, United States of America; 2 Pritzker School of Medicine, University of Chicago, Chicago, Illinois, United States of America; 3 Maestría en Investigación Clínica, Escuela Superior de Medicina, Instituto Politécnico Nacional, Ciudad de México, Mexico; 4 División Académica de Ciencias de la Salud, Universidad Juárez Autónoma de Tabasco, Villahermosa, Tabasco, Mexico; 5 Jurisdicción Cunduacán, Secretaría de Salud de Tabasco, Cunduacán, Tabasco, Mexico; 6 Saint Luke School of Medicine, Alliant International University, Ciudad de México, Mexico; 7 Institute for Risk Assessment Sciences, Utrecht University, Utrecht, The Netherlands; 8 Hospital Regional de Cunduacán, Secretaría de Salud de Tabasco, Cunduacán, Tabasco, Mexico; 9 CETis No. 70, SEP, Villahermosa, Tabasco, Mexico; 10 Hospital Regional de Alta Especialidad Dr. Gustavo A. Rovirosa Pérez, Villahermosa, Tabasco, Mexico; 11 Facultad de Medicina, Universidad Nacional Autónoma de México (UNAM), Ciudad de México, Mexico; 12 Section of Infectious Disease, University of Chicago Comer Children’s Hospital, Chicago, Illinois, United States of America; 13 Departamento de Infectología e Inmunología, Instituto Nacional de Perinatología, Secretaría de Salud, Ciudad de México, Mexico; University of Colorado Anschutz Medical Campus: University of Colorado - Anschutz Medical Campus, UNITED STATES OF AMERICA

## Abstract

**Background:**

Women living in leishmaniasis-endemic zones are regularly exposed to the sandfly vector in their environments. While case series and laboratory evidence consistently suggest transplacental transmission of *Leishmania* parasites with deleterious maternal-fetal effects, this issue has received insufficient attention, particularly in areas where the predominant *Leishmania* species is mainly associated with cutaneous disease.

**Methodology/principal findings:**

We conducted an exploratory cross-sectional study in a highly endemic zone of Tabasco, Mexico, enrolling 53 women with singleton term deliveries between April 2018 and April 2020. Placental PCR was positive in 18 (34%) participants. Buccal swabs were positive in 11 (21.2%) of 52 newborns. Immunofluorescence confirmed intracellular amastigotes within macrophages near the vascular endothelium of PCR-positive placentas, with no surrounding inflammatory infiltration. Sequencing revealed homology to *Leishmania mexicana*, *L. amazonensis*, or *L. panamensis*. Birthweight percentile was modestly lower in the PCR-positive group (predicted mean 53.8% vs. 56.5%, p = 0.76), while small for gestational age showed a non-significant trend toward higher prevalence among PCR-positive cases (prevalence ratio = 2.06, 95% CI: 0.32–13.39, p = 0.45).

**Conclusions/significance:**

Subclinical, dynamic transmission of *Leishmania* parasites typically associated with cutaneous disease was detected in this endemic zone. The presence of *L. mexicana* in human placentas was confirmed by immunofluorescence and sequencing, without an associated inflammatory response. These findings highlight the potential of CL-associated *Leishmania* species to reach the placenta and buccal mucosa of newborns, warranting further epidemiological investigation into the consequences of vertical transmission in regions with endemic CL.

## Author summary

Leishmaniasis is a parasitic infection mainly known for causing skin ulcers (cutaneous leishmaniasis) or severe internal disease (visceral leishmaniasis). While congenital transmission of *Leishmania* has been documented in visceral disease, the potential for cutaneous species to cross the placenta has received less attention. We studied 53 pregnant women living in a highly endemic region of Tabasco, Mexico, and found that one-third harbored *Leishmania* DNA in their placentas, while about one-fifth of their newborns showed positive buccal swabs. Importantly, the women showed no clinical signs of cutaneous leishmaniasis, indicating that subclinical infection can still allow parasites to reach the placenta. We confirmed the presence of the parasite in placental tissue using immunofluorescence microscopy and DNA sequencing; the sequences matched *Leishmania* species associated with cutaneous disease, and species-specific sequencing confirmed *Leishmania mexicana*. The parasite was found inside immune cells (macrophages), without causing visible inflammation, suggesting the parasite can persist silently in placental tissue. These findings are important for women of reproductive age living in or travelling to cutaneous leishmaniasis-endemic regions, as they and their newborns may be at risk of subclinical exposure to the parasite.

## Introduction

Leishmaniasis is a protozoal infection caused by *Leishmania* parasites and is traditionally classified into visceral leishmaniasis (VL) and cutaneous leishmaniasis (CL). VL is characterized by hepatosplenomegaly, fever, anemia, and weight loss, whereas CL manifests as granulomatous or ulcerative skin lesions. Globally, an estimated 900,000 to 1.6 million new cases occur each year [1]. Transmission occurs through the bite of infected sandflies, primarily *Lutzomyia* in the Americas and *Phlebotomus* in the Old World. In endemic regions, up to one in 50 sandflies may harbor *Leishmania* [2]. Laboratory studies suggest female sandflies have short adult lifespans, typically fewer than 20 days, though natural populations may live considerably longer [3]. Mature females require a blood meal during each oviposition cycle; with inter-bite intervals averaging approximately 6 days, they typically acquire parasites from only a small number of blood meals over their lifespan [3]. Although cutaneous lesions are recognized in humans and animals, they occur relatively infrequently, raising the question of potential subclinical infection [4].

Treatment of leishmaniasis, particularly during pregnancy, is challenging. The only compound with demonstrated efficacy and an acceptable safety profile in pregnant women with visceral leishmaniasis is liposomal amphotericin B [5]. Experience in pregnant women with cutaneous leishmaniasis is scarce, although liposomal amphotericin B has recently been used successfully [6]. Physical methods such as thermotherapy or cryotherapy may also be safe during pregnancy for cutaneous leishmaniasis, although their effects on the fetus remain unknown; immunotherapeutic and vaccine approaches remain experimental [7]. None is established for the prevention or treatment of subclinical or congenital infection, which underscores the importance of recognizing at-risk groups such as pregnant women living in endemic regions.

The fact that *Leishmania* parasites have been detected in the blood of both symptomatic patients and asymptomatic individuals living in endemic areas of CL [8] suggests that CL infection is not limited to skin alone. Animal models provide additional evidence supporting this observation: *Leishmania* parasites may disseminate to distant tissues [9], including the placenta, as demonstrated in a murine model [10]. This finding challenges the traditional dichotomy in which VL is viewed as strictly systemic and CL as strictly cutaneous. The placenta presents a unique opportunity to investigate subclinical infection due to its high degree of vascularization and routine expulsion after delivery. It receives approximately 0.5 litres of blood per minute, meaning that the total blood volume of an adult circulates through it every ten minutes [11]. This extensive circulation enables continuous exchange of nutrients, immune cells, and potentially pathogens between the mother and fetus. During passage through the placenta, some parasites may cross the barrier, a phenomenon well described for malaria, Chagas’ disease, and toxoplasmosis, which has been linked to adverse maternal and infant outcomes including anemia, low birth weight, pre-eclampsia, restricted fetal growth, and infant mortality [12,13].

Given these parallels, it is plausible that *Leishmania* may also cross the placenta, leading to adverse pregnancy outcomes [14]. Evidence supporting vertical transmission of *Leishmania* has been described in VL, where both symptomatic and asymptomatic mothers have been shown to transmit the parasite to their offspring [5,15,16]. However, studies on the vertical transmission of CL-associated species remain scarce, although their findings are suggestive. In a Brazilian cohort of pregnant women with CL (n = 26), 10.5% experienced preterm birth and 10.5% had stillbirths, both associated with exuberant lesions [17]. Additionally, *L. mexicana* was identified as the cause of visceral leishmaniasis in a 6-month-old infant from Tabasco, the first visceral case reported in this region where cutaneous disease predominates, indicating that a species typically associated with cutaneous leishmaniasis can visceralize in early childhood [18]. Nevertheless, the prevalence of *Leishmania* infection (clinical or subclinical) among women of reproductive age, and the effects of locally endemic CL-associated strains such as *L. mexicana* on birth outcomes, remain largely unknown. Given that suboptimal fetal growth is six times more prevalent in developing regions [19], many of which overlap with leishmaniasis-endemic zones, it is important to determine whether any relationship exists between them.

Diseases caused by protozoa (malaria, toxoplasmosis, Chagas disease, and visceral leishmaniasis) are well-documented causes of adverse fetal development, prompting public health efforts to enhance screening, diagnosis, and treatment [20,21]. In contrast, leishmaniasis, particularly CL-associated strains, has not received the same level of attention as a potential threat to perinatal health. No study has systematically investigated the presence of *Leishmania* in placental tissue from women in CL-endemic regions, alongside the buccal mucosa of their newborns, or its potential association with adverse perinatal outcomes.

We conducted an exploratory cross-sectional study in a highly endemic zone of Tabasco, Mexico, with three specific aims: (1) to determine the frequency of placental infection by *Leishmania* sp. by PCR analysis, hypothesizing that subclinical infection by CL-associated species could be identified in the placentas of women living in endemic zones; (2) to ascertain the frequency of *Leishmania* sp. infection in the oral mucosa of newborns, hypothesizing that vertical transmission could occur either hematogenously or via parasite-harboring amniotic fluid reaching the buccal mucosa of the fetus; and (3) to explore the association of placental *Leishmania* infection with fetal growth, hypothesizing that infection would be associated with fetal growth restriction.

## Materials and methods

### Ethics statement

The study protocol was approved by the Institutional Review Boards of the Instituto Nacional de Perinatología (#2017-3-116), the *Secretaría de Salud de Tabasco* (#INV/2133/PCI/1116), and Indiana University (#1905780741). All participants provided written informed consent during prenatal consultations. As the legal guardians of the neonates, mothers granted written consent for their newborn children’s participation, covering placental sampling, buccal swab, and urine collection performed at delivery. A red dot label was placed on clinical records after providing consent to identify study participants as placental donors at hospital arrival for delivery. All participants received standard-of-care prenatal and delivery care at no cost, as provided within the public health system, at a secondary or tertiary care hospital in the study area. PCR-positive results were not treated as clinical cases of leishmaniasis. The detection of parasite DNA is not equivalent to active clinical infection; furthermore, the drugs used to treat clinical leishmaniasis carry significant risks including hepatotoxicity and nephrotoxicity that outweigh likely benefits in the absence of clinical disease. Quarterly technical reports were submitted to health authorities in Tabasco to ensure follow-up monitoring of mothers and infants for potential CL lesions or clinical signs of vertical transmission.

### Study design and setting

**Fig 1:**
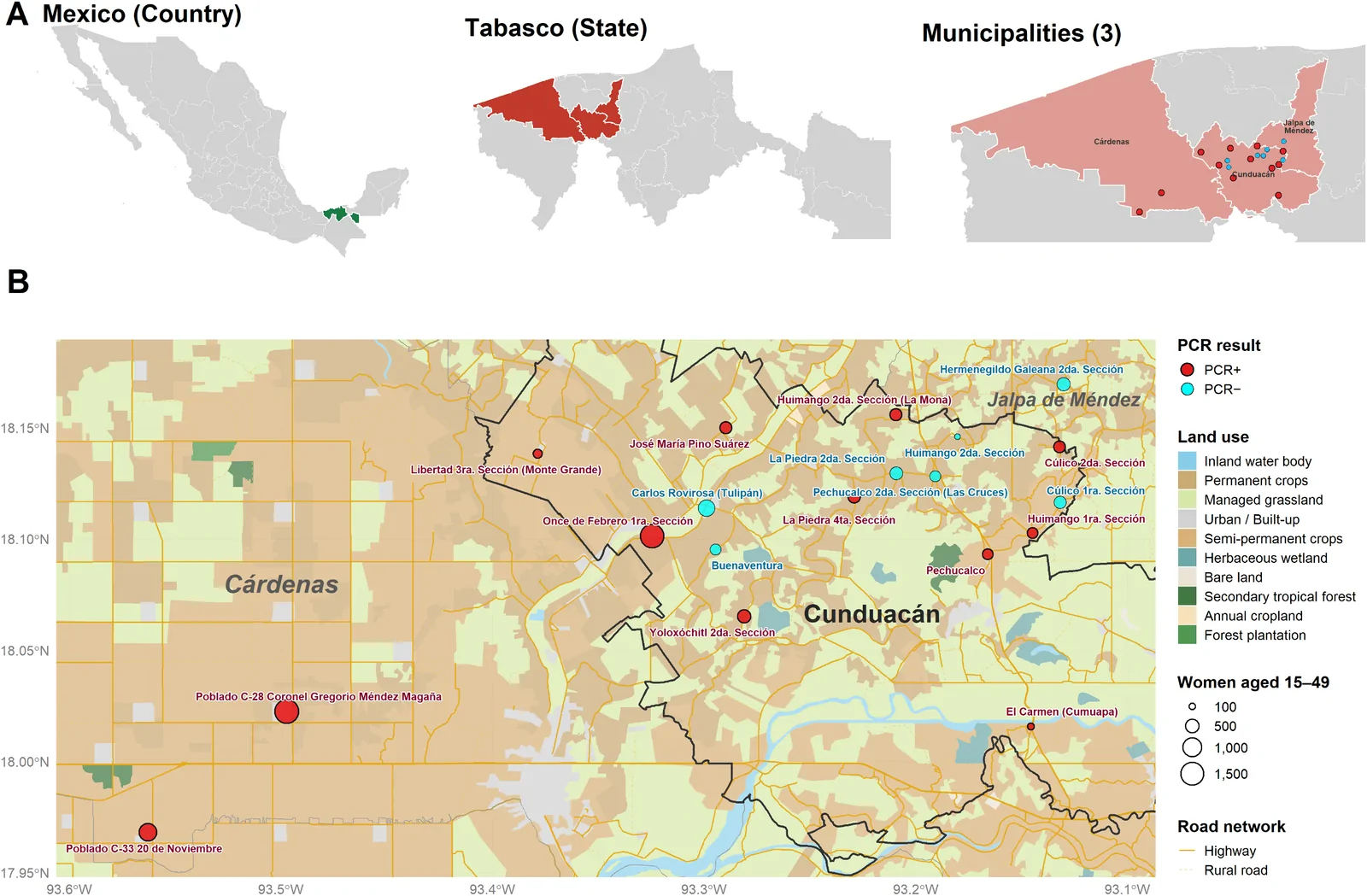
Regional distribution of placental leishmaniasis (PCR-positive) cases and land use in Cunduacán and neighboring municipalities (Tabasco, Mexico, 2017–2019). **(A)** Geographic location of the study area within Mexico. **(B)** Spatial distribution of sampled localities by placental PCR status. Each circle represents a sampled locality centroid from the INEGI 2020 Geostatistical Framework, the smallest geographic unit reported in publicly available census data; circle size is proportional to the number of women aged 15–49 years per locality (INEGI Census 2020). Red circles indicate localities with at least one PCR-positive placental sample; cyan circles indicate localities where all samples were PCR-negative. Map data sources: Panel A (country and state outlines): Natural Earth 1:10m cultural vectors, public domain (https://www.naturalearthdata.com/downloads/10m-cultural-vectors/; licence: https://www.naturalearthdata.com/about/terms-of-use/). Panels A–B (municipal boundaries): INEGI Municipal Geostatistical Framework, 2020 (https://en.www.inegi.org.mx/app/biblioteca/ficha.html?upc=889463807469). Panel B (land use and vegetation): INEGI Land Use and Vegetation Series VII, 1:250,000, 2018 (https://en.www.inegi.org.mx/app/biblioteca/ficha.html?upc=889463842781). Water bodies in Panel B are shown as the inland-water areas of the same INEGI Land Use and Vegetation layer (Series VII). Panel B (road network): INEGI National Road Network, 2020 (https://en.www.inegi.org.mx/app/biblioteca/ficha.html?upc=702825292805). All INEGI datasets and shapefiles are published under Mexico’s open-data licence (https://en.www.inegi.org.mx/inegi/terminos.html).

We conducted a cross-sectional exploratory study in Tabasco, Mexico, a State in the south-east of the country encompassing some of the highest CL-endemic zones in Mexico. Highly endemic zones, defined as areas with more than 200 cases per 10,000 inhabitants in the preceding five years, were selected for sampling. Study zones were selected on the basis of the availability of National Health Services (*Secretaría de Salud*) facilities and accessibility by road (Fig 1).

Three secondary-level regional hospitals located in Cárdenas, Comalcalco, and Paraíso, and two tertiary-level hospitals (*Hospital de la Mujer* and *Hospital Rovirosa*) where women with complicated pregnancies are referred, were invited to participate. Community health houses (*casas de salud*) managed by the Tabasco Health Services were used as recruitment centers for pregnant women beginning at their first prenatal visit. Participating women then delivered at the corresponding hospital facility.

#### Participant recruitment.

Women were eligible for inclusion if they: resided in a highly endemic CL zone, were in their first trimester of pregnancy, carried a singleton fetus, and received prenatal care at one of the participating *Secretaría de Salud* facilities. Women were excluded if they had multiple fetuses, a molar pregnancy, a history of toxoplasmosis, malaria, rubella, HIV, or exposure to other known teratogens, or a fetal trisomy. A total of 53 pregnant women meeting the inclusion criteria were recruited between April 2018 and April 2020. Enrollment was restricted to singleton pregnancies since multiple gestation constitutes a high-risk pregnancy with distinct antenatal surveillance and management, and because the INTERGROWTH-21st birthweight-percentile standard used to classify small-for-gestational age is derived from and validated for singleton pregnancies [22].

### Data collection

Nurses, doctors, and students involved in the care of pregnant women in the endemic area received training on project objectives, sample collection procedures, and participant communication. Quarterly refresher sessions were conducted to address questions and reinforce the training.

### Medical records

Data on maternal age, reproductive history (parity, prior miscarriages, prior stillbirths, prior Caesarean section), medical history (obesity, smoking, alcohol use, and drug use), prenatal clinical and laboratory findings (hypertension, gestational diabetes, hemoglobin), and previous or current *Leishmania* skin lesions were obtained by chart review of hospital records by researchers. Anemia in pregnancy was defined as a hemoglobin concentration in blood lower than 11.0 g/dL [23]. Screening for subclinical or placental *Leishmania* infection in prior pregnancies was not available, as it is not part of routine antenatal care.

### Perinatal outcomes

Newborn data obtained from medical records included estimated gestational age in weeks (as determined by the best available dating method, with ultrasound-based dating and delivery-time registration in agreement), anthropometric measurements including birthweight (g) and crown-heel length (cm). Birthweight percentile for gestational age and sex was calculated using the INTERGROWTH-21st standard [22]. Small-for-gestational age (SGA) was defined as birthweight below the 10th percentile for gestational age and sex.

### Placental samples

At delivery, trained medical personnel collected four placental tissue samples of approximately 1 cm³ each from every participant for histological analysis and PCR testing. Placentas were sampled from four adjacent locations, two at the border and two close to the umbilical cord. Samples intended for histological analysis were stored in 1% formaldehyde (Sigma-Aldrich, USA). Samples intended for DNA extraction, PCR, or sequencing were stored in RNA Protect Tissue Reagent (Qiagen, Hilden, Germany).

### Buccal samples

Trained medical personnel collected a buccal swab from each newborn within the first few minutes after delivery and before their first feeding. Buccal swabs were preserved in 300 μL of Cell Lysis Solution (Gentra Puregene Buccal Cell Kit, Qiagen, Hilden, Germany). All laboratory testing was performed at the Instituto Nacional de Perinatología in Mexico City.

### Laboratory analysis

#### DNA extraction.

Placental tissue was disrupted using a Tissue Lyser LT (Qiagen, Hilden, Germany) with steel beads (Qiagen, Hilden, Germany) for 20 min at 40 Hz. DNA extraction was then performed using a QIAamp DNA Mini Blood Kit (Qiagen, Hilden, Germany) following the manufacturer’s protocol. Buccal swabs were processed using a Gentra PureGene Buccal Cell Kit (Qiagen, Hilden, Germany) per the manufacturer’s recommendations.

#### PCR amplification.

**Fig 2:**
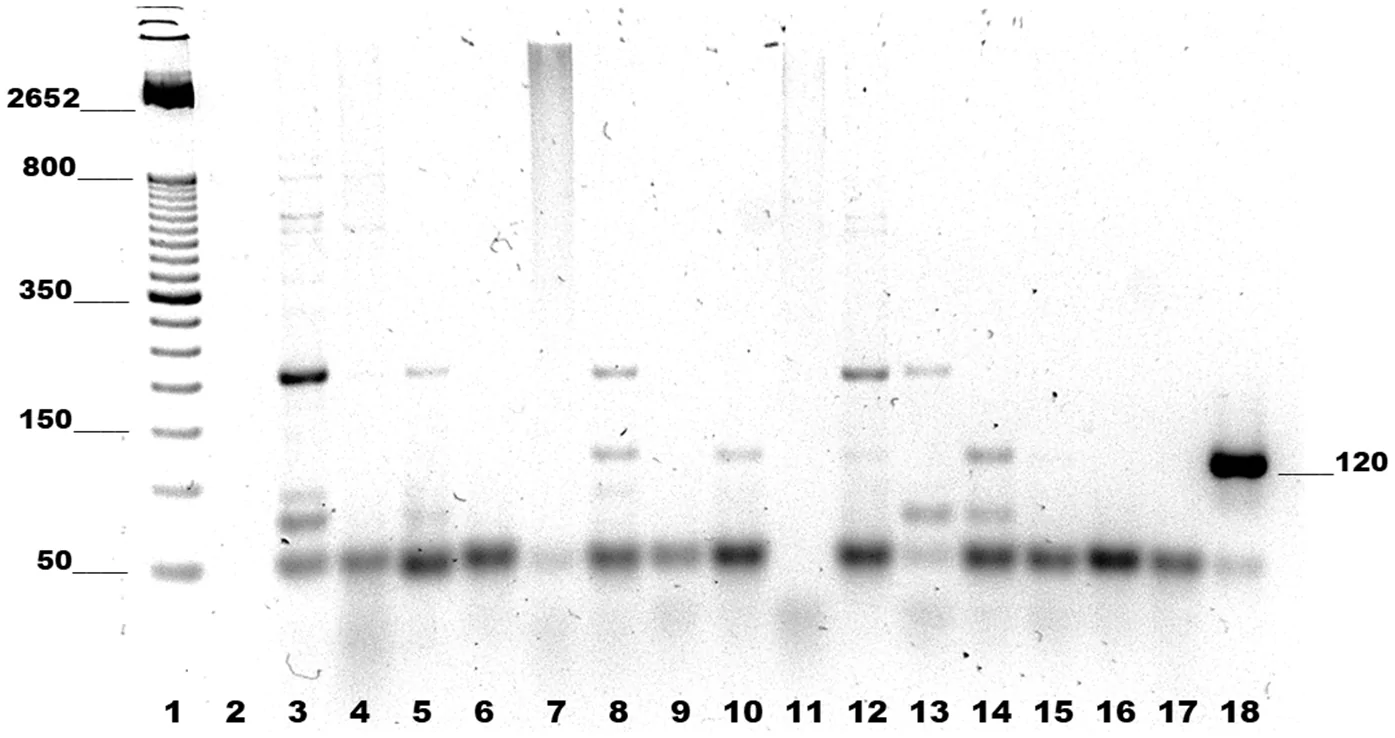
Representative agarose gel electrophoresis of placental samples subjected to endpoint PCR amplification. Lane 1: molecular weight marker; lane 2: reagents (no-template) control; lanes 3–16: paired border and inner placental samples; lane 17: *Trypanosoma cruzi* DNA as a negative amplification control; lane 18: *Leishmania mexicana* DNA as positive control, showing the expected 120-bp band amplified with JW11–JW12 primers. Lanes 3–4: term labor sample from a non-endemic zone, without the 120-bp band. Lanes 5–8 and 13–16: term pregnancy samples from endemic zone; the 120-bp band is present in lanes 8 and 14 (placenta border). Lanes 9–12: term pregnancy samples from two women with high-risk pregnancies who delivered at a tertiary-level hospital; the 120-bp band is visible in lanes 10 and 12. Note: This gel is representative of the PCR analysis and does not display all 18 PCR-positive participants. Densitometric analysis was performed on the 120-bp bands in lanes 8, 10, 12 and 14, and on the positive-control band in lane 18; the band-analysis report is provided in S1 File.

DNA amplification for *Leishmania* sp. detection used the kinetoplast minicircle primers JW11 (forward, 5’-CCTATTTTACACCAACCCCCAGT-3’) and JW12 (reverse, 5’-GGGTAGGGGCGTTCTGCGAAA-3’). These primers amplify an approximately 120-bp fragment of the minicircle kDNA of *L. major*, which contains 10,000 copies per parasite [24]. End-point PCR used Taq PCR Master Mix (Qiagen, Germany) in a final volume of 25 μL. Cycling conditions were: initial denaturation at 95°C for 10 min; 40 cycles of denaturation at 95°C for 1 min, annealing at 58°C for 30 sec, and elongation at 72°C for 30 sec; and final extension at 72°C for 10 min (Fig 2).

#### Controls.

Positive controls included *Leishmania mexicana* (MNYC/BZ/62/M379) and *Leishmania mexicana* (MHOM/MX/01/Tab3), as well as genomic DNA from *Trypanosoma cruzi* (*ninoa mexicana*), *Toxoplasma gondii* strain RH ATCC 50174D, and *Plasmodium falciparum* Welch Strain 3D7 Genomic DNA ATCC PRA-405D. Negative controls consisted of reactions without a DNA template. The integrity of the extracted DNA was verified by 1% agarose gel electrophoresis and confirmed through amplification of the human β-globin gene.

#### Sequencing.

Two sequencing protocols were followed to confirm the identity of amplified DNA. PCR-amplified bands were visualised by agarose gel electrophoresis, extracted with a QIAquick Gel Extraction Kit (Qiagen, Germany), and sent to the Instituto de Biotecnología of the Universidad Nacional Autónoma de México (UNAM) for sequencing. Secondary sequencing was also performed for the internal transcribed spacer 1 (ITS1) and 5.8S ribosomal gene regions, a validated molecular target for *Leishmania* species identification [25,26], at the Indiana University-Bloomington Global Environmental Health Laboratory. Bioinformatic analyses compared sequences with GenBank database records via the Basic Local Alignment Search Tool (BLAST) at the National Center for Biotechnology Information (NCBI; http://www.ncbi.nlm.nih.gov).

#### Histology.

For immunofluorescence detection of *Leishmania* in placental tissues, tissue sections were fixed on glass slides. Antibodies were purified from a patient with diffuse cutaneous leishmaniasis from Comalcalco, Tabasco, using a Protein G column (Sigma-Aldrich, St. Louis, MO, USA). Antibodies were labeled using the Mix-n-Stain CF 488A Antibody Labeling Kit (Sigma-Aldrich) per the manufacturer’s instructions and used at a 1:50 dilution. Counterstaining was performed using a 5 μg/mL solution of propidium iodide (Thermo Fisher Scientific, Baltics, UAB) for 3 min. Antibody purification, titration, and specificity testing against the amastigote stage are described in S1 Text and S1 Fig. To preserve antigenicity for immunofluorescence, sections destined for immunofluorescence were not stained with haematoxylin and eosin or with Giemsa. As a morphological reference for the appearance of intracellular amastigotes by Giemsa staining, a section from the footpad of a mouse experimentally infected with *L. mexicana* is provided (S2 Fig).

### Data analysis

Descriptive statistics for maternal sociodemographic, reproductive, prenatal, and medical history characteristics, and infant outcomes are presented as n (%) for categorical variables, mean ± SD for normally distributed continuous variables, and median (IQR) for non-normally distributed continuous variables. Given the small and unequal group sizes, between-group comparisons used Welch’s t-test or Wilcoxon rank-sum test for continuous variables, and chi-square or Fisher’s exact test for categorical variables, as appropriate. Statistical significance was defined as p < 0.05.

The prevalence ratio (PR) of small for gestational age per placental PCR positivity was estimated using a modified Poisson regression model (log link) and robust standard errors with 95% confidence intervals (95% CI) [27]. To examine the association between placental PCR status and birthweight percentile, a fractional logit regression model (quasibinomial family with logit link) was fitted and presented as odds ratios (OR) with 95% CI. Predicted mean birthweight percentiles with 95% CI were estimated from the model and displayed graphically alongside observed data. Due to the small sample size, multivariable adjustment was not performed; all regression estimates are therefore unadjusted (crude).

The enrollment of 53 participants are the total number of women who met eligibility criteria and provided consent over the study period (April 2018–April 2020); no formal *a priori* power calculation was performed given the exploratory nature of the study.

For variables with missing data, complete-case analysis was used. Hemoglobin values were available for 34 of 53 participants (64%); reasons for missingness were not recorded. Analysis of buccal PCR status was based on 52 newborns, as one swab was not received. Birthweight percentile and SGA classification were based on 52 newborns (35 PCR-negative, 17 PCR-positive), as data needed for the percentile were unavailable for one PCR-positive newborn. No additional sensitivity analyses were pre-planned given the exploratory study design.

All analyses were conducted in R (version 4.6.0) through R Studio (version 2026.07.1). The source code for this manuscript, statistical analyses, and project documentation is available through the online repository: https://github.com/javimangal/leishmania-placental. The dataset supporting this study is publicly available [28].

## Results

### Study population and PCR analysis

Most (51 of 53; 96%) of the study participants delivered at the Regional Hospital of Cunduacán; the remainder gave birth at Villahermosa hospital sites (n = 2). Placental PCR was positive in 18 (34%) participants and negative in 35. The geographic distribution of PCR-positive cases across sampled localities is shown in Fig 1; positive cases were distributed across 13 out of 19 (68.4%) sampled localities.

The amplification of a 120-bp fragment, which appears as a unique product when DNA is amplified from *L. mexicana* reference strains as well as from regional isolates, was used as the criterion for a positive result. Both the internal (near the umbilical cord) and external placental regions underwent successful PCR analysis for all 53 maternal participants. Among the 18 PCR-positive participants, positivity was detected in both placental regions in 5, exclusively in the internal region in 8, and exclusively in the external region in 5. By band intensity, the internal region was positive in 13 samples (8 intense, 5 faint) and the external region in 10 samples (5 intense, 5 faint); densitometric analysis of the representative gel is provided in S1 File. The agarose gel in Fig 2 shows a representative subset of these samples rather than all 18 PCR-positive cases; the uncropped original gel image is provided in S1 Raw Gel.

Of the 52 newborn buccal swabs successfully tested for DNA integrity, 11 (21.2%) tested positive by endpoint PCR. However, only 3 cases showed matching positive results for both the buccal swab and placental samples; 8 newborns with PCR-positive buccal swabs were born from mothers with PCR-negative placentas.

### Confirmation of parasite presence by immunofluorescence

**Fig 3:**
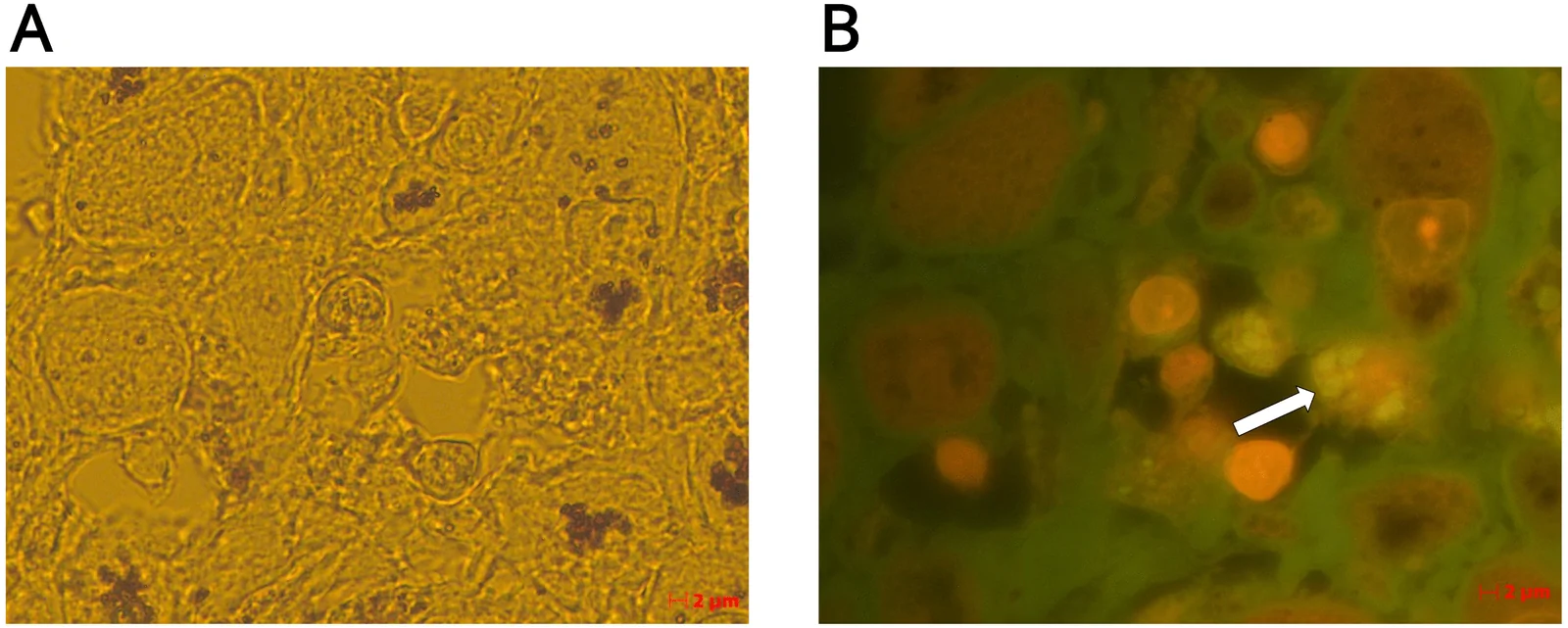
Immunofluorescence of a PCR-positive placenta (basal decidua). Both panels show the same field. **(A)** Brightfield image. **(B)** The same field after staining with a CF 488A–conjugated anti-*Leishmania* antibody (green) purified from a patient with diffuse cutaneous leishmaniasis, with propidium iodide counterstain (red). The arrow indicates a macrophage containing fluorescent intracellular amastigotes. Scale bar, 2 μm. Original magnification, × 1000.

Histological analysis was performed on eight placentas (three PCR-positive and five PCR-negative). In all participants, histological examination revealed features consistent with term placentas. In PCR-positive placentas, macrophages containing fluorescent structures resembling intact amastigotes were observed. These parasitized fluorescent macrophages (pfMØs) were detected on both the maternal and fetal sides, ranging from the syncytiotrophoblast to the chorionic villi. The pfMØs were specifically located at the margins of fetal sinusoids and on the endothelium of the basal decidua (Fig 3). Fluorescent macrophages were also identified within the characteristic reticular stroma of the basal plate and intermediate villi, extending from the syncytiotrophoblast. In all cases, fluorescence was observed inside the macrophages without surrounding inflammatory cell infiltration, suggesting the absence of an active inflammatory response. The fluorescent intracellular structures corresponded in size and morphology to *Leishmania* amastigotes, whose appearance by Giemsa staining is illustrated in a section from the footpad of a mouse experimentally infected with *L. mexicana* (S2 Fig). No pfMØs were detected in PCR-negative placentas.

### Confirmation and identification of *Leishmania* by sequencing

Three placentas with intense bands on the PCR agarose gel were selected for band elution and sequencing. The 120-bp forward product of the first sample showed 90% homology to *L. mexicana* (Z11556.1); this participant was a woman aged 30–34 at 39 weeks of gestation with hypertension who delivered a newborn in the 90^th^ weight percentile. The second sample showed 87% homology to *L. amazonensis* (EU370874); this participant was a woman aged 20–24 in her fourth pregnancy with a prior miscarriage who delivered at 40 weeks with a newborn also in the 90^th^ weight percentile. A third sample showed homology to *L. panamensis* (M87314.1); this participant was a woman aged 25–29 with a history of miscarriage and gestational diabetes who delivered a newborn in the 95^th^ weight percentile. All three samples presented sequence homology to a region within the minicircle of kinetoplast DNA. Separate amplification using a *L. mexicana* primer targeting the ITS1 and 5.8S ribosomal gene regions confirmed the presence of *L. mexicana* in placental samples (GenBank accession KU177477.1; 617 bp; 93–99% homology to reference strain M379). This ITS1 assay additionally identified *L. mexicana* (97–99% homology) in two further placental samples, distinct from the three described above. The first corresponded to a woman younger than 20 years in her second pregnancy, who had experienced a pregnancy loss at 13.1 weeks of gestation in her first pregnancy and delivered a newborn at the 10^th^ weight percentile at 38 weeks of gestation. The second corresponded to a woman aged 20–24 years in her third pregnancy, who delivered a newborn at the 70^th^ weight percentile at 39 weeks of gestation.

### Maternal and infant characteristics by *Leishmania* PCR status

Table 1 presents the comparison of maternal and infant characteristics by placental PCR status. Most participants (69.8%) were between 20 and 35 years of age. Mean (SD) maternal age was 24.6 ± 6.6 years overall and did not differ significantly between PCR groups (p = 0.16). Most participants had delivered at least once before (11.3% had ≥ 3 prior deliveries). Average parity did not differ between groups (p = 0.27). None of the participants presented with CL lesions. None reported tobacco, alcohol, or illicit drug use during pregnancy.

**Table 1:** Comparison of maternal and infant characteristics by *Leishmania* PCR result in placental samples.

Variable	Total (n = 53)	PCR negative (n = 35)	PCR positive (n = 18)	p-value
***Maternal age***				
Maternal age (yrs), mean ± SD	24.6 ± 6.6	23.7 ± 6.2	26.4 ± 7.1	0.18 ^a^
< 20 yrs, n (%)	12 (22.6)	10 (28.6)	2 (11.1)	0.32 ^b^
20–34 yrs, n (%)	37 (69.8)	23 (65.7)	14 (77.8)	
≥ 35 yrs, n (%)	4 (7.5)	2 (5.7)	2 (11.1)	
***Reproductive history***				
Parity, median (IQR)	2.0 (1.0–3.0)	2.0 (1.0–3.0)	2.5 (1.0–3.0)	0.36 ^c^
Primiparas, n (%)	22 (41.5)	15 (42.9)	7 (38.9)	1.00 ^d^
Multiparas, n (%)	31 (58.5)	20 (57.1)	11 (61.1)	
Prior miscarriage (multiparas), n/N (%)	4/30 (13.3)	1/19 (5.3)	3/11 (27.3)	0.13 ^d^
Prior stillbirth (multiparas), n (%)	1 (3.2)	1 (5.0)	0 (0.0)	1.00 ^d^
Prior caesarean section (multiparas), n/N (%)	2/30 (6.7)	1/19 (5.3)	1/11 (9.1)	0.26 ^d^
***Maternal clinical & laboratory findings***				
Hypertension, n (%)	7 (13.2)	4 (11.4)	3 (16.7)	0.68 ^d^
Gestational diabetes, n (%)	2 (3.8)	1 (2.9)	1 (5.6)	1.00 ^d^
Haemoglobin (g/dL), mean ± SD (n = 34)	12.3 ± 1.3	12.0 ± 1.3	13.0 ± 1.1	**0.04** ^**a**^
Anaemia (Hb < 11.0 g/dL), n/N (%)	10/34 (29.4)	8/24 (33.3)	2/10 (20.0)	0.68 ^d^
***Infant sex***				
Male, n (%)	26 (49.1)	20 (57.1)	6 (33.3)	0.15 ^d^
Female, n (%)	27 (50.9)	15 (42.9)	12 (66.7)	
***Infant PCR***				
Buccal swabs, n positive (%), n/N	11/52 (21.2)	8/34 (23.5)	3/18 (16.7)	0.73 ^d^

^a^Welch’s two-sample t-test. ^b^ Chi-squared test. ^c^ Wilcoxon rank-sum test. ^d^ Fisher’s exact test.

Among multiparas, prior miscarriage was more common in the PCR-positive group than in the PCR-negative group (3/11 [27.3%] vs. 1/20 [5.0%]; p = 0.13), although this difference was not statistically significant. The groups did not differ significantly with respect to prior stillbirth or prior Caesarean section. The proportion of male newborns was lower in the PCR-positive group (6/18, 33.3%) than in the PCR-negative group (20/35, 57.1%), though this difference was not statistically significant (p = 0.18).

### Perinatal outcomes

Table 2 presents the comparison of perinatal outcomes by PCR status. Gestational age and birthweight did not differ significantly between groups (p = 0.19 and p = 0.60, respectively). Crown-heel length was slightly lower in the PCR-positive group (median 50.5 vs. 52.0 cm), though this difference was not significant (p = 0.28). Five-minute Apgar scores were uniformly 9 in both groups, with no infant scoring below 7. SGA was observed in 2/17 (11.8%) of the PCR-positive group versus 2/35 (5.7%) of the PCR-negative group (p = 0.59). The crude prevalence ratio for small for gestational age of PCR-positive cases compared to PCR-negative was PR = 2.06 (95% CI: 0.32–13.39; p = 0.450).

**Table 2:** Comparison of perinatal outcomes by *Leishmania* PCR result in placental samples.

Variable	Total (n = 53)	PCR negative (n = 35)	PCR positive (n = 18)	p-value
***Perinatal outcomes***				
Gestational age (wks), median (IQR)	39.0 (38.0–40.0)	39.0 (39.0–40.0)	39.0 (38.0–40.0)	0.19 ^a^
Birthweight (g), median (IQR)	3320.0 (3000.0–3500.0)	3400.0 (3100.0–3500.0)	3220.0 (2862.5–3552.5)	0.60 ^a^
Crown-heel length (cm), median (IQR)	51.0 (49.5–53.0)	52.0 (50.0–53.0)	50.5 (49.0–52.0)	0.28 ^a^
***Birthweight classification***				
Weight percentile, median (IQR)	55.0 (40.0–80.0)	60.0 (45.0–80.0)	50.0 (40.0–70.0)	0.87 ^a^
SGA (< 10th percentile), n/N (%)	4/52 (7.7)	2/35 (5.7)	2/17 (11.8)	0.59 ^b^
***Apgar score***				
5-min. Apgar score, median (IQR)	9.0 (9.0–9.0)	9.0 (9.0–9.0)	9.0 (9.0–9.0)	0.61 ^a^
5-min. Apgar score < 7, n (%)	0 (0.0)	0 (0.0)	0 (0.0)	–

^a^Wilcoxon rank-sum test; ^b^ Fisher’s exact test.

Abbreviations: SGA = small-for-gestational age.

### Birthweight percentile by PCR status

**Fig 4:**
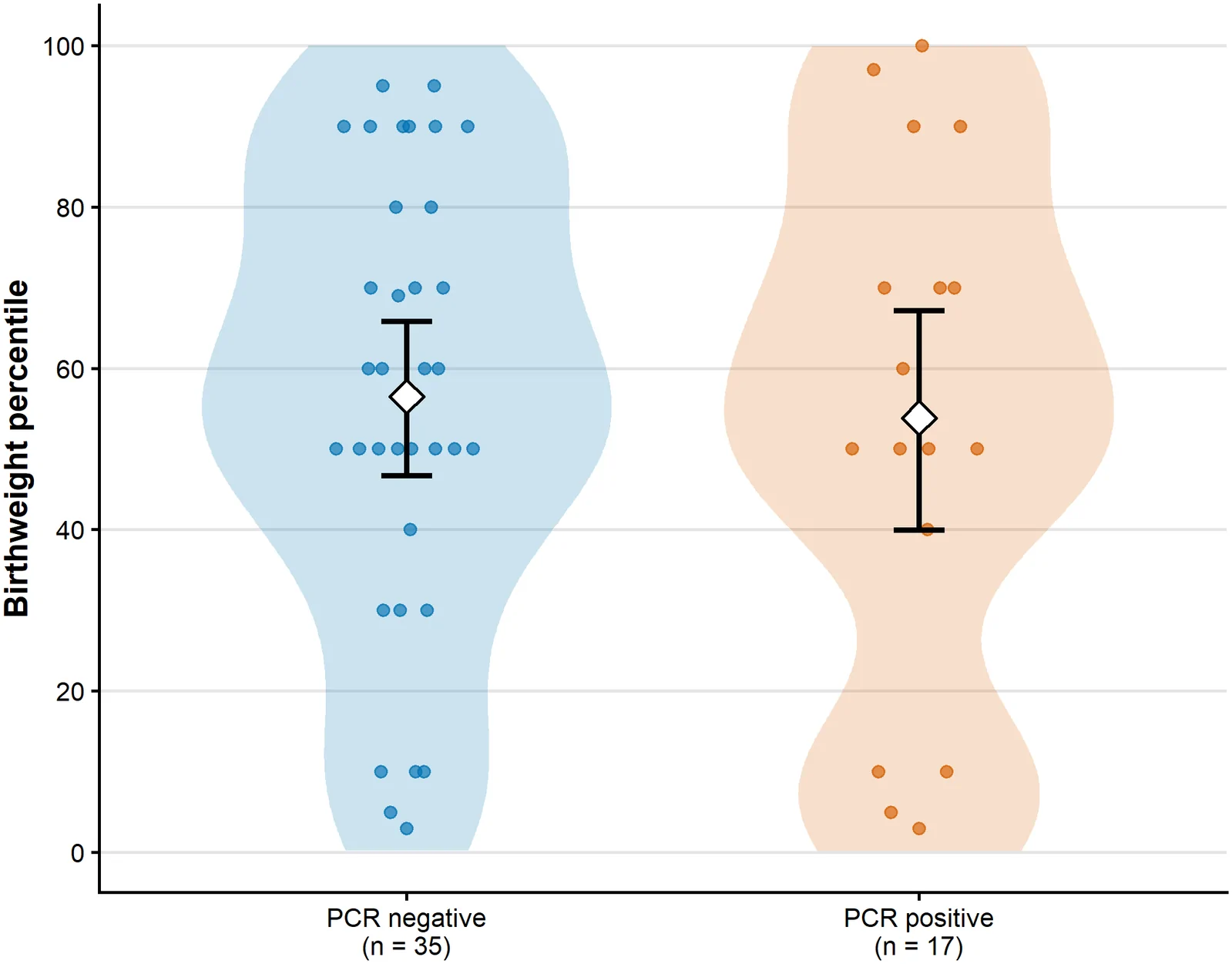
Birthweight percentile by placental PCR status. Points show individual observations; shaded areas correspond to the density distribution. White diamonds and bars show fractional logit predicted means with 95% confidence intervals.

Placental PCR positivity was associated with a modest, non-significant reduction in birthweight percentile (Fig 4). The fractional logit model estimated lower odds of a higher birthweight percentile in PCR-positive compared to PCR-negative women (OR 0.9, 95% CI 0.45–1.78; p = 0.76). Model-predicted mean birthweight percentiles were 56.5% (95% CI 46.7–65.8) in the PCR-negative group and 53.8% (95% CI 39.9–67.1) in the PCR-positive group, corresponding to an absolute difference of 2.7 percentile points (95% CI -14.3–19.6).

## Discussion

The present findings confirm the presence of *Leishmania mexicana*, a species traditionally associated with cutaneous lesions, in a relatively high proportion of human placentas from CL-endemic regions of Mexico. One-third of placentas tested positive by PCR, and parasitized macrophages were confirmed by immunofluorescence in PCR-positive placentas, without an associated inflammatory response.

Congenital transmission of *Leishmania* is well documented for VL and is associated with adverse pregnancy outcomes, including fetal loss, low birth weight, and the subsequent development of visceral leishmaniasis in infants [29,30]. For CL, such transmission has been poorly documented. Our results, together with the recent demonstration of vertical transmission of *L. donovani* in a mouse model of VL [15], add to the growing body of evidence that *Leishmania* parasites from both visceral and cutaneous species can reach the placenta.

For other hematological protozoans with congenital transmission such as *Toxoplasma*, *Trypanosoma*, and *Plasmodium*, adverse pregnancy effects have been predominantly associated with the first pregnancy, suggesting a protective effect of maternal immunity in subsequent pregnancies [20,31]. In contrast, in our study perinatal complications were more common in multiparas (specifically prior miscarriage), although this difference was not statistically significant. Because subclinical or congenital *Leishmania* infection is not screened for in routine antenatal care, the *Leishmania* status of these women’s previous pregnancies is unknown, and we cannot determine whether PCR-positive participants had a previously affected fetus. We propose that environmental exposure to infected sandflies in this endemic area may lead to subclinical maternal infection and subsequent parasite dissemination into the placenta during the current gestation, consistent with the lack of cutaneous lesions across all participants.

Compared with the high proportion of the population harboring *Leishmania* parasites in this endemic zone, the prevalence of clinical disease is relatively low. Several factors may explain this discrepancy, including the low pathogenicity of circulating *Leishmania* strains, low parasite load, or individual host susceptibility [31]. Additionally, circulating strains in this zone have been previously reported to undergo apoptotic destruction *in vitro* at physiological temperature (37°C) [32]; however, within the complex *in vivo* environment, the parasite’s ability to survive may differ, potentially because rescue molecules may influence its persistence. Consistent with persistence *in vivo*, the amastigotes detected by immunofluorescence retained their characteristic oval morphology; because loss of viability in this species is accompanied by a transition from an oval to a spherical form [32], the preserved oval shape is compatible with structurally intact, potentially viable parasites rather than residual parasite debris.

Whether DNA detected in tissue reflects true colonization or contamination has been central to the debate over the putative placental microbiome, where the organ’s low microbial biomass, the sensitivity of DNA-based assays, and the recovery of unexpected taxa have cast doubt on culture-independent evidence [33,34]. Our findings are less vulnerable to this ambiguity: immunofluorescence localized intact amastigotes inside placental macrophages rather than free DNA, and sequencing of the amplified product confirmed homology to Leishmania—together indicating that the PCR signal arose from whole, intracellular parasites and not from spurious or contaminating DNA.

The detection of parasites by PCR does not distinguish between live and dead parasites, and this study did not include parasite cultures from placental samples, both of which are limitations of this work. A notable histological feature observed in this study, compared with infections caused by *T. cruzi* and *T. gondii*, was the absence of a host-stimulated immune response [35]. Unlike those protozoa, immune cells were not observed in the vicinity of mononuclear cells harboring *Leishmania* parasites, consistent with the parasite’s well-characterized ability to evade immune responses. In a mouse model of VL, IFN signal activation and cellular immune suppression have been shown to accompany placental *L. donovani* infection [15], further supporting the concept that *Leishmania* parasites may create an immunologically permissive environment within the placenta.

The detection of parasite DNA in one-fifth of newborn oral cavity samples suggests either contact with amniotic fluid harboring parasites or infection via the hematological route. In a mouse model infected with *L. mexicana* via the footpad, the parasite disseminated to all vascularized organs studied, including the salivary glands [9], supporting the plausibility of hematogenous transmission to the newborn’s oral mucosa. Notably, 8 of the 11 buccal PCR-positive newborns were born from mothers whose placental samples tested PCR-negative. This discordance has several plausible explanations. First, placental infection may have been present but undetected: a low parasite burden together with the focal, irregular distribution of amastigotes in infected tissue means that the small fragments sampled for PCR may not have contained parasites, producing false-negative placental results despite true infection, a sampling limitation also well recognized for amastigote detection in bone-marrow aspirates in visceral leishmaniasis. Second, the newborn oral cavity may have been exposed to parasites independently of detectable placental infection, for example through contact with parasite-harboring amniotic fluid, maternal blood, or genital-tract secretions during passage through the birth canal, so that intrapartum contamination cannot be excluded. Disentangling true transplacental transmission from peripartum acquisition will require longitudinal mother–infant sampling with quantitative and more extensive placental analysis. A longitudinal follow-up of this cohort could provide valuable insights into potential long-term effects on the offspring’s immune tolerance [5].

The minor sequence variations observed among the sequenced samples suggest the existence of genetically diverse circulating strains. Future studies should analyze the protozoal microbiome of the placenta and compare the genetic sequences of parasites detected in placental tissue with those from cutaneous lesions, to identify potentially non-pathogenic strains. For *T. cruzi* [36] and *Plasmodium* [37], specific parasite strains involved in congenital transmission have been identified; it is plausible that a similar pattern exists for *Leishmania*.

Reproductive-age women living in or travelling to tropical regions endemic for CL represent a high-risk population for adverse pregnancy outcomes and subclinical congenital transmission. In non-endemic areas, they may also serve as a potential source of *Leishmania* congenital transmission. The full consequences of such transmission remain unknown, as congenital CL has not been previously suspected. A key aspect to consider is the potential impact on central immune tolerance development in offspring exposed *in utero*, which warrants further investigation.

Limitations of this study include the cross-sectional exploratory design precluding causal inferences. The sample size was small, reducing statistical power to detect differences in perinatal outcomes; expansion of the original recruitment target was rendered infeasible by the COVID-19 pandemic. PCR positivity does not confirm the presence of viable parasites as PCR detects DNA from both live and dead organisms, which may overestimate the prevalence of active infection. The absence of parasite cultures is a further limitation in this respect. Hemoglobin data were available for only 34 of 53 participants (64%), limiting the assessment of anemia as an outcome; the reasons for missing data were not recorded. The generalizability of these findings to other CL-endemic regions should be approached with caution. Sandfly vector species, circulating *Leishmania* strains, and host immune responses may vary substantially across geographic areas, and these results may not be representative of all endemic populations.

## Conclusion

*Leishmania* DNA was detected in one-third of placentas from women living in CL-endemic zones with term pregnancies, confirming that dynamic subclinical transmission occurred below clinical expression levels. The presence of *Leishmania mexicana* in placental tissue was confirmed by direct immunofluorescence and sequencing. Notably, inflammatory infiltrates were not observed surrounding the mononuclear cells harboring the parasites, suggesting a clinically silent infection. Parasite DNA was also detected in the buccal mucosa of one-fifth of newborns. These findings call for increased awareness of the potential for CL-associated *Leishmania* species to undergo vertical transmission, with implications for maternal and neonatal health surveillance in endemic regions.

## Supporting information

S1 Text Purification and validation of the anti-*Leishmania* human antibody. IgG was purified from the serum of a patient with diffuse cutaneous leishmaniasis using a Protein G column and conjugated with CF 488A. Reactive fractions were identified by titration, and the working dilution and specificity were established as summarized in S1 Fig.
(PDF)

S1 Fig Validation of the anti-*Leishmania* human antibody used for immunofluorescence. (TIF)

S2 FigGiemsa-stained section from the footpad of a mouse experimentally infected with *Leishmania mexicana.*Representative fields at increasing magnification (×10, ×40, ×100) showing intracellular amastigotes within mononuclear cells, provided as a morphological reference for the appearance of amastigotes by Giemsa staining.(TIF)

S1 File Densitometric band analysis of the agarose gel shown in Fig 2. Band-analysis report exported from AlphaView 3.4.0.0 for the gel image in Fig 2 (16-bit grayscale). Bands correspond to the 120-bp product in lanes 8, 10, 12 and 14, and to the positive-control band (*Leishmania mexicana* M379) in lane 18. Band intensity is not quantitative and was not associated with perinatal outcome or histological findings.
(PDF)

S1 Raw Gel Original agarose gel image for Fig 2. Uncropped, original image of the endpoint-PCR agarose gel shown in Fig 2, captured with AlphaView software after GelRed staining. Lane identities, the molecular-weight ladder, and the 120-bp positive-control band are annotated, and wells not included in Fig 2 are marked with an X. This image is also the source of the densitometric analysis in S1 File.
(PDF)
