# Supplementary material for: Placentas from healthy women living in a cutaneous leishmaniasis endemic region in Mexico harbor Leishmania mexicana parasites: Potential implications for pregnancy and neonatal outcomes
Source: PLoS Negl Trop Dis. 2026 Sep 15;20(9):e0014367. doi: 10.1371/journal.pntd.0014367 (PMC13588509; doi:10.1371/journal.pntd.0014367)
Supplement: Supplementary file 1 [file pntd.0014367.s001.pdf]

S1 Text. Purification and validation of the anti-*Leishmania* human antibody used for immunofluorescence.

Serum source and antibody purification

Immunoglobulins were purified from the serum of a patient with diffuse cutaneous leishmaniasis residing in Comalcalco, Tabasco, the same endemic zone from which the study participants were recruited. Thus, the antibody repertoire would reflect exposure to the *Leishmania* species circulating locally. Immunoglobulin G was isolated by affinity chromatography on a Protein G column (Sigma-Aldrich, St. Louis, MO, USA) and collected as sequential elution fractions. Purified immunoglobulin was subsequently conjugated with CF 488A using the Mix-n-Stain CF 488A Antibody Labeling Kit (Sigma-Aldrich) according to the manufacturer's instructions.

Screening of elution fractions

Each elution fraction was screened by immunofluorescence against *Leishmania mexicana* promastigotes, the species prevalent in the study zone and the stage that can be maintained in culture. Fractions yielding specific fluorescent labelling of the parasite were retained for further characterisation; fractions 6 to 8 were reactive; these were pooled for the specificity testing described below.

Titration and selection of the working dilution

The reactive antibody preparation was titrated across serial dilutions (1:10, 1:50, 1:100, 1:200 and 1:500) and examined at increasing magnification. Specific labelling of parasites was retained across the range tested, with progressive loss of signal intensity at higher dilutions. A dilution of 1:50 was selected as the working concentration, providing strong specific signal with acceptable background, and was used for all subsequent staining of placental tissue (**S1 Fig**, panel B).

Specificity against the amastigote stage

Because the parasite stage expected in tissue is the amastigote rather than the promastigote, specificity was confirmed on imprints prepared from the footpad of *Leishmania*-infected BALB/c mice. Imprints were stained in parallel under four conditions: buffer alone; buffer with propidium iodide counterstain and no primary antibody; the CF 488A–conjugated anti-*Leishmania* antibody alone; and the conjugated antibody with propidium iodide counterstain. Omission of the primary antibody produced nuclear counterstaining without green signal, whereas the conjugated antibody specifically labelled intracellular amastigotes. A Giemsa-stained imprint processed in parallel provided the morphological reference (**S1 Fig**, panel A). Triton X-100 was included in the washes and in the primary antibody incubation to permeabilise the cells.

Application to placental tissue

Once specificity and working dilution were established, the validated antibody was applied to placental tissue sections, with propidium iodide (5 µg/mL, 3 min; Thermo Fisher Scientific,

Baltics, UAB) as counterstain. Parasitized macrophages identified in PCR-positive placentas are shown in **Fig 3** of the main text.

Staining strategy and antigen preservation

To preserve antigenicity for immunofluorescence, placental sections destined for immunofluorescence were not stained with haematoxylin and eosin or with Giemsa. Separate placental sections were stained with Giemsa to provide a histological correlate (**S2 Fig**). The size and morphology of amastigotes observed in the Giemsa-stained placental sections were compared with those seen in the immunofluorescence-stained placentas and were found to be comparable in both dimensions and shape, supporting the identification of the fluorescent intracellular structures as intact amastigotes.
