# Supplementary material for: Placentas from healthy women living in a cutaneous leishmaniasis endemic region in Mexico harbor Leishmania mexicana parasites: Potential implications for pregnancy and neonatal outcomes
Source: PLoS Negl Trop Dis. 2026 Sep 15;20(9):e0014367. doi: 10.1371/journal.pntd.0014367 (PMC13588509; doi:10.1371/journal.pntd.0014367)
Supplement: Supplementary file 2 [file pntd.0014367.s004.pdf]

PCR 070218.tif

Creation Date:

03/07/2026 15:28:41

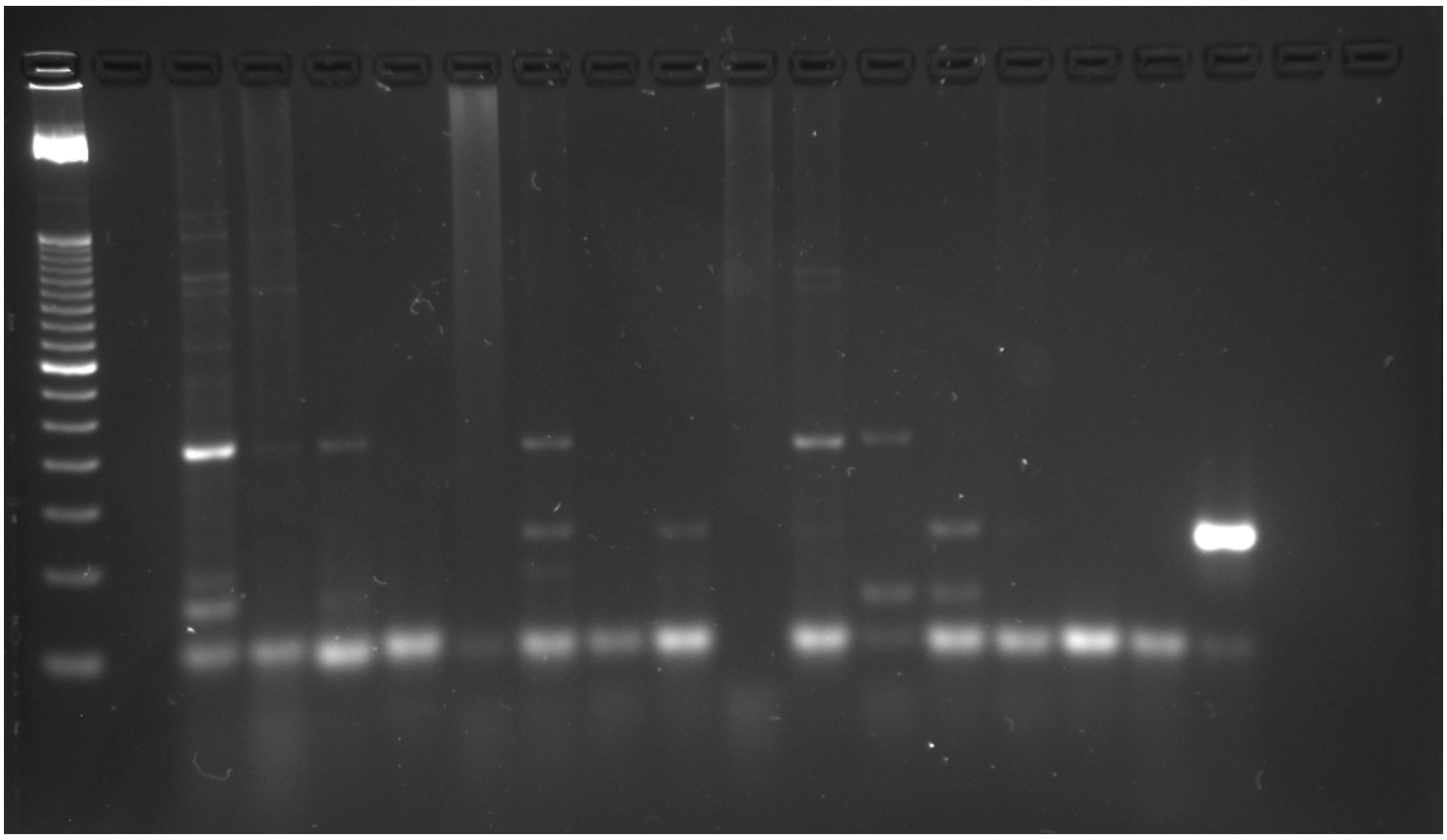

Original Image

Acquisition Information

Product name	AlphaView
Software version	3.4.0.0
Acquisition Date	02/07/2026
Acquisition Time	14:59:59
Modified	No
Image Width	991
Image Height	570
Image Type	Grayscale
Bits Per Pixel	16
Protocol Name	n/a
Cooler Temperature	Cooler n/a
Acquisition Information	n/a

Analysis Information

Band	Sum	Area	Average	Median

1	6,333,690	333	19,020.09	19,076
2	7,945,079	455	17,461.71	17,492
3	2,270,400	132	17,200.00	17,236
4	8,393,507	384	21,858.09	21,941
5	56,777,994	1,035	54,857.97	65,535

Report End
