# Supplementary material for: Placentas from healthy women living in a cutaneous leishmaniasis endemic region in Mexico harbor Leishmania mexicana parasites: Potential implications for pregnancy and neonatal outcomes
Source: PLoS Negl Trop Dis. 2026 Sep 15;20(9):e0014367. doi: 10.1371/journal.pntd.0014367 (PMC13588509; doi:10.1371/journal.pntd.0014367)
Supplement: Supplementary file 3 [file pntd.0014367.s005.pdf]

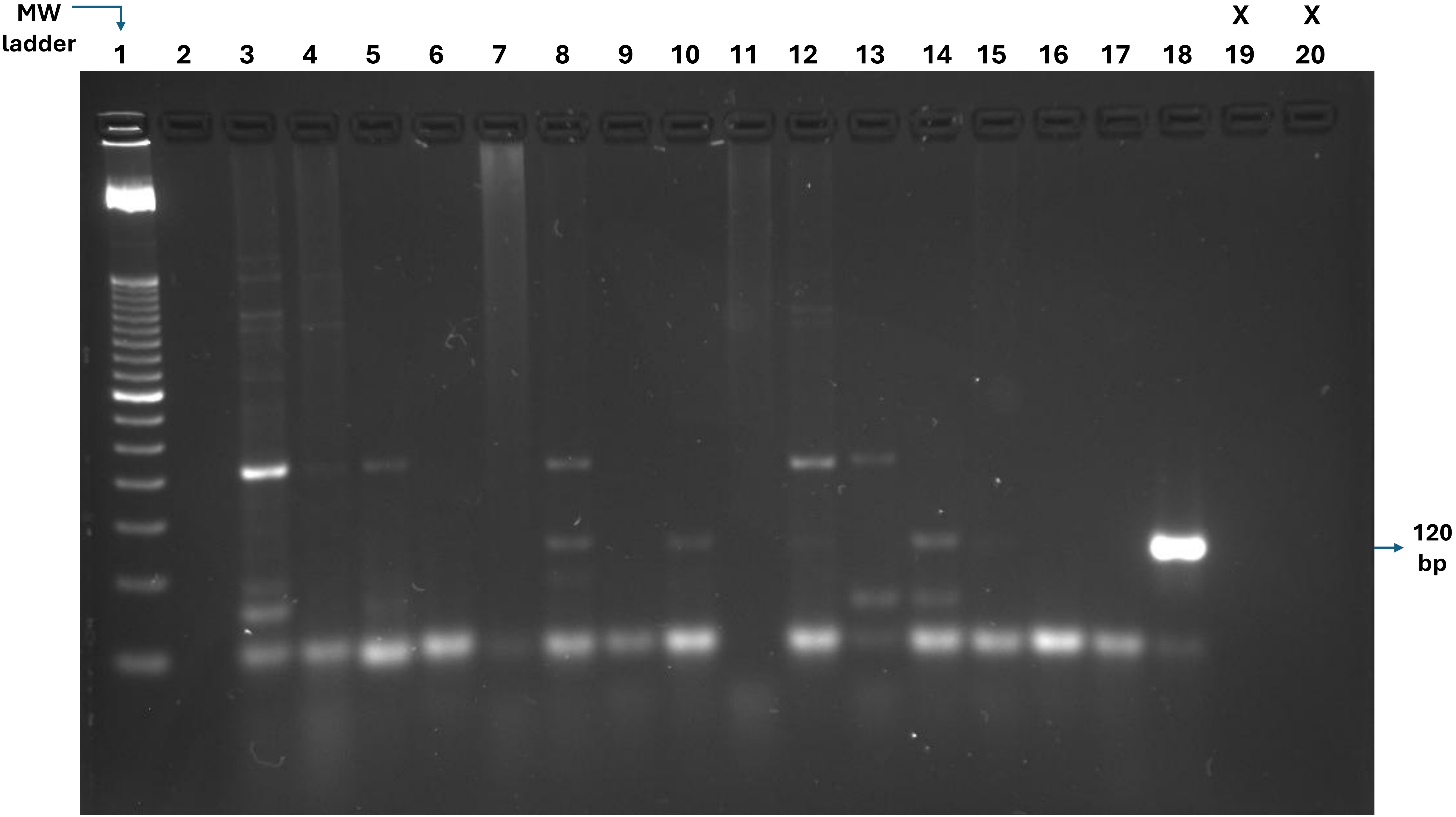
MW
ladder
X
X
1
2
3
4
5
6
7
8
9
10
11
12
13
14
15
16
17
18
19
20
120
bp
