# Supplementary material for: Placentas from healthy women living in a cutaneous leishmaniasis endemic region in Mexico harbor Leishmania mexicana parasites: Potential implications for pregnancy and neonatal outcomes
Source: PLoS Negl Trop Dis. 2026 Sep 15;20(9):e0014367. doi: 10.1371/journal.pntd.0014367 (PMC13588509; doi:10.1371/journal.pntd.0014367)
Supplement: Supplementary file 4 [file pntd.0014367.s006.docx]

**Andrés F. Henao-Martínez, Academic Editor**
**Shaden Kamhawi and Paul Brindley, Editors-in-Chief**
**PLOS Neglected Tropical Diseases**

Dear Dr. Henao-Martínez and Editors,

We thank the editors and both reviewers for their constructive evaluation of our manuscript, “*Placentas from healthy women living in a cutaneous leishmaniasis endemic region in Mexico harbor* Leishmania mexicana *parasites: Potential implications for pregnancy and neonatal outcomes*.” We have revised the manuscript accordingly and respond to each point below. For each point, the reviewer’s comment are shown in *italics*, followed by our response. All page and line numbers cited in this letter refer to the **revised clean manuscript PDF version** submitted with this revision; the same changes are marked in the tracked-changes version.

A preprint of this work is now publicly available on medRxiv (Armijos et al. 2026). Raw gel images are provided as Supporting Information. We have additionally deposited data for reproduction in the Harvard Dataverse, as described at the end of this rebuttal letter. Other purely technical submission-form items (manuscript source format, figure file formats, trademark symbols) are handled in the submission form and figure uploads as instructed, and are noted here only where they affect the manuscript.

# Reviewer #1

We thank the reviewer for these comments, which helped us improve the reporting and clarity of the manuscript.

**Comment 1.**

*Authors are suggested to add treatment options for leishmaniasis which is missing in the introduction section, recent studies have suggested potential gene KO strategies for vaccine candidates and various plant-based therapies are in use for decades. Authors are suggested to cite these publications: PMID: 40196396, PMID: 33528770, https://link.springer.com/article/10.1007/s11756-024-01656-y*

**Response.** We agree that the Introduction benefits from a treatment overview and have added a paragraph summarizing first-line therapy relevant to pregnancy: liposomal amphotericin B for visceral leishmaniasis and, for cutaneous disease due to *L. mexicana*, its combination with physical methods, although supporting data are scarce. We also note that immunotherapeutic and vaccine approaches remain experimental (Torres-Guerrero et al. 2017). We carefully evaluated the three suggested references. They report experimental antileishmanial compounds and plant-derived extracts evaluated against *Leishmania donovani* (visceral disease) in murine models. While valuable in drug-discovery research, they are specific to visceral disease and to preclinical candidates, and do not address the cutaneous-disease context of our study population; we therefore cite a comprehensive leishmaniasis review with explicit coverage of pregnancy, cutaneous disease, and *L. mexicana*, which we believe better serves the reader at this point in the Introduction (Introduction, p. 3, lines 16–25).

**Comment 2.**

*The authors have included women with a singleton fetus pregnancy. Please provide a rationale for the same. Also the positive cases have any history of prior fetus with positive Leishmaniasis? If not, what could have been the cause of the congenital transmission this time?*

**Response.** We restricted enrollment to singleton pregnancies to avoid the confounding effect of multiple gestation on the perinatal growth outcomes, and because the INTERGROWTH-21st birthweight-percentile standard used to define small-for-gestational age is derived from and validated for singleton pregnancies. We have made this rationale explicit (Methods, Participant Recruitment, p. 4, lines 95–98).

Regarding prior affected pregnancies: *Leishmania* status was not assessed in any previous pregnancy of the participants, because no screening program for subclinical or congenital *Leishmania* infection exists in this population; we therefore cannot report whether PCR-positive participants had a previously affected fetus, and we now state this. The most parsimonious explanation for the placental positivity we observed is ongoing environmental exposure to infected sandflies in this highly endemic zone, leading to subclinical maternal infection and parasite dissemination to the placenta, consistent with the absence of clinical cutaneous lesions in all participants (Discussion, p. 14, lines 335–343). We have also added a note to the Methods stating that screening for subclinical or placental *Leishmania* infection in prior pregnancies was not available, as it is not part of routine antenatal care (Methods, Medical Records, p. 6, lines 127–129).

**Comment 3.**

*Figure 3, appears to be a representative image from one patient sample collected across the placenta and other controls run on agarose gel. It is suggested to quantify the patient samples’ Agarose gel bands with respect (densitometric analysis), to the positive control lane 17, and possible quantification should be shown across other samples. Also how do the light and dark bands of 120bp gene in patient samples represent the disease outcome and how does that correlate with histopathology or other data? This must be discussed in the results and discussion.*

**Response.** We have performed densitometric analysis of the 120-bp bands, and the unaltered band-analysis report is provided as S1 File. The analysed bands are now identified in the Fig 2 legend: the 120-bp bands in lanes 8, 10, 12 and 14, and the positive-control band in lane 18 (Fig 2 legend, p. 7).

We did not normalize the sample bands to the positive-control lane, and we would respectfully explain why. The positive-control band is saturated: its median intensity reaches 65,535, the maximum value representable in the 16-bit image. Its true intensity is therefore unknown and greater than the value recorded, so any ratio computed against it would be biased by an unknown amount. Repeating the band analysis with independently drawn band boundaries changed the apparent intensity of this saturated band by roughly 40%, whereas the sample bands varied by less than 6% and retained their rank order. We have therefore reported the measured band intensities without normalization and treat the positive control as a qualitative reference rather than a quantitative calibrator, which we believe is the more defensible reading of these data.

We also clarify the lane assignment to remove ambiguity: in the original gel (now Fig 2 after renumbering), *Trypanosoma cruzi* DNA (negative amplification control) is in lane 17 and *L. mexicana* M379 (positive control) is in lane 18; the Fig 2 legend callouts have been set accordingly (p. 7).

Regarding the relationship between band intensity and disease outcome or histopathology: because end-point PCR of the high-copy kinetoplast minicircle target is not quantitative, band intensity reflects amplification efficiency rather than parasite burden and cannot be reliably equated with clinical severity. Consistent with this, we found no systematic association between band intensity and perinatal outcome or histological findings. We have therefore tempered any interpretation of band intensity throughout the manuscript and now state this explicitly where the densitometric data are presented: the Fig 2 legend identifies the analysed bands and directs the reader to the report (p. 7), the Results point to the densitometric analysis of the representative gel (p. 9, lines 243–245), and the S1 File caption states that band intensity is not quantitative and was not associated with perinatal outcome or histological findings (p. 16, lines 444–448). The intense/faint descriptors are retained in the Results only as the qualitative reading of the gel from which the samples for densitometry and sequencing were selected.

**Comment 4.**

*Figure 4, A and B image legends do not match the image description. What does image 4B represent if it’s the same image, show the arrow with macrophage and intracellular parasite. Scale bar is required in the images. The parasite inside the macrophage should be quantified or the infected macrophages corresponding to Figure 4. Hand E stain of the placenta should also be incorporated.*

**Response.** We have revised this figure (now Fig 3 after renumbering) and its legend so that the panels correspond exactly: panel A is the brightfield image of the basal-decidua field, and panel B is the same field stained with the CF 488A–conjugated anti-*Leishmania* antibody (green) and propidium iodide counterstain (red). We have corrected the antibody descriptor, which previously read “FITC-conjugated,” to “CF 488A–conjugated” to match the Methods. An arrow now indicates the macrophage containing fluorescent intracellular amastigotes, and a calibrated scale bar (2 µm; original magnification ×1000) has been added directly from the microscopy software so that the scale matches the image exactly.

To document antibody specificity, we have added S1 Fig, which shows the validation workflow: confirmation of reactivity against the amastigote form in *L. mexicana*-infected BALB/c footpad imprints (with a no-primary-antibody control, a propidium-iodide counterstain, and a parallel Giemsa stain), together with the dilution series from which the 1:50 working dilution was selected. The full narrative serum source, Protein G purification, screening of elution fractions against cultured *L. mexicana* promastigotes, titration, and specificity testing is provided in S1 Text. Giemsa-stained fields demonstrate that the fluorescent structures correspond in size and morphology to amastigotes.

Regarding histology: immunofluorescence and conventional histochemical stains cannot be applied to the same section, because the histochemical processing degrades the antigens required for immunofluorescence. No hematoxylin–eosin (H&E) sections are therefore available. The absence of an inflammatory infiltrate surrounding the parasitized macrophages was assessed directly on the immunofluorescence preparations, in which the parasitized fluorescent macrophages were not accompanied by surrounding inflammatory cells. To document the morphology of the parasite, a Giemsa-stained section from the footpad of a mouse experimentally infected with *L. mexicana* is provided in S2 Fig as a reference for the appearance of intracellular amastigotes; the rationale for omitting H&E (preservation of antigenicity for immunofluorescence) is now stated explicitly in the Methods (Histology, p. 8, lines 193–198), where S1 Text, S1 Fig and S2 Fig are also cited. Given the focal and irregular distribution of amastigotes, the confirmatory immunofluorescence was not designed for formal density quantification; we report the parasitized macrophages observed and have framed this finding as qualitative and confirmatory rather than quantitative (Results, p. 9–10, lines 252–265). The absence of an inflammatory infiltrate around the parasitized cells is stated in the Results (p. 10, lines 261–264) and revisited in the Discussion (p. 15, lines 365–373).

# Reviewer #2

We thank the reviewer for the supportive assessment and the specific, helpful suggestions.

## Introduction

**Page 3, line 47.**

*“Protozoan infections (malaria, toxoplasmosis, Chagas’ disease, and visceral leishmaniasis)”. I suggest changing to “Diseases caused by protozoa (malaria, toxoplasmosis, Chagas, and visceral leishmaniasis)” or write the scientific names of the protozoa (not the disease) in parentheses.*

**Response.** Rephrased to “Diseases caused by protozoa (malaria, toxoplasmosis, Chagas disease, and visceral leishmaniasis)” (Introduction, p. 3, line 57).

## Materials and Methods

**Page 4, line 71.**

*The font size in the caption for Figure 1 is too small.*

**Response.** The small caption was embedded in the Fig 1 image file; it has been removed from the image in the figure-generating script, and its content now appears in the manuscript figure legend at standard text size.

**Page 6, Figure 2.**

*In my opinion, this figure is unnecessary; the methodology for collecting samples from the placenta and buccal mucosa of newborns is clear and already described in the paragraph (lines 123-135).*

**Response.** We agree and have removed the sampling-schematic figure (former Figure 2 of the original submission), relying on the Methods description of placental and buccal sampling. All references to it have been removed and the subsequent figures renumbered.

## Results

**Page 9, line 209.**

*Most (37 of 53; 96%) of the study participants….; 37 out of 53 participants is 68.9%*

**Response.** We thank the reviewer for catching this error. The intended statement was that 51 of 53 participants (96%) delivered at the Regional Hospital of Cunduacán, with the remaining 2 delivering at Villahermosa sites. This has been corrected (Results, p. 9, lines 232–233).

**Page 9, lines 218–221.**

*“In five patients, both the internal and external placental regions were positive; the internal region tested positive in 13 samples (8 showing intense bands, 5 faint bands), while the external region tested positive in 10 samples (5 intense, 5 faint; Figure 3).” Figure 3 is a representative gel sample from the PCR analysis, as it shows the results of some placental samples and not all 18 positive samples. I suggest also changing the text to/or something similar to; “PCR positivity was observed for both the internal and external placental regions in 5 samples, exclusively for the internal region in …. samples, and solely for the external region in …. samples (Figure 3)”*

**Response.** We have clarified in both the legend and the Results text that the gel (now Fig 2) is a representative sample rather than a display of all 18 positive cases, and we have made the regional breakdown explicit: PCR positivity was observed in both placental regions in 5 participants, exclusively in the internal region in 8, and exclusively in the external region in 5, consistent with the 18 PCR-positive participants. We have retained the intense/faint band-intensity counts (internal region: 13 positive, 8 intense and 5 faint; external region: 10 positive, 5 intense and 5 faint) because these are the source values for the densitometric analysis requested by Reviewer 1 (point 3) (Results, p. 9, lines 237–246; Fig 2 legend, p. 7).

## Discussion

**Page 14, lines 317–320.**

*“The detection of parasites by PCR does not distinguish between live and dead parasites, and this study did not include parasite cultures from placental samples.” Are there histological sections stained with hematoxylin-eosin? It would be interesting to show if there was inflammatory cellular infiltrate around the parasitized cells. It would also be interesting to macerate part of the infected tissues and inoculate them into culture medium or even infect laboratory animals to assess the viability/infectivity of amastigotes revealed by immunofluorescence. This could indicate that the congenital infection occurs from amastigotes present in the placenta, supporting the plausibility of hematogenous transmission to the newborn’s oral mucosa.*

**Response.** No hematoxylin–eosin sections are available, because the antigen-preserving fixation required for immunofluorescence is incompatible with H&E processing; the absence of an inflammatory cellular infiltrate around the parasitized cells was therefore assessed directly on the immunofluorescence preparations, in which the parasitized macrophages were not surrounded by inflammatory cells, and this is now stated explicitly in the Results and Discussion (see Reviewer 1, point 4, for the stain rationale). For morphological reference, a Giemsa-stained section from the footpad of a mouse experimentally infected with *L. mexicana*, illustrating the appearance of intracellular amastigotes, is provided in S2 Fig. We agree that culture and infectivity assays (tissue maceration into culture medium or animal inoculation) would be valuable to confirm amastigote viability and infectivity; these were not feasible within the present cross-sectional study, in which placental tissue was fixed or preserved for DNA extraction rather than for culture. The absence of parasite cultures is acknowledged as a limitation, alongside the fact that PCR cannot distinguish live from dead parasites (Discussion, Limitations, p. 15–16, lines 408–413), and we agree that culture and infectivity assays are the necessary next step. We would add one indirect line of evidence bearing on viability: the amastigotes detected by immunofluorescence retained their characteristic oval morphology, whereas loss of viability in this species is accompanied by a transition from an oval to a spherical form (Galindo-Sevilla 2022); the preserved oval shape is therefore compatible with structurally intact, potentially viable parasites rather than residual parasite debris. This point has been added to the Discussion (p. 14, lines 351–355).

**Page 14, lines 332–336.**

*“Notably, 8 of the 11 buccal PCR-positive newborns were born from PCR-negative placenta mothers, suggesting that the placenta is not the only potential route and that contamination at the time of delivery cannot be excluded. A longitudinal follow-up of this cohort could provide valuable insights into potential long-term effects on the offspring’s immune tolerance” Isn’t it more likely that the parasite load was low or the distribution of amastigotes in the placenta was irregular, and the tissue sample collected for PCR did not contain amastigotes, even though the pregnant woman’s placenta was infected? In bone marrow aspirations from patients with visceral leishmaniasis, these aspects can lead to variable results in the identification of amastigotes. What other infection mechanisms could occur, including those during childbirth? Authors have mentioned it. Discuss this further.*

**Response.** We agree and have revised this passage. We now explain that the discordance between buccal-positive newborns and PCR-negative placentas may reflect a false-negative placental result, because a low parasite burden together with the focal, irregular distribution of amastigotes means that the small tissue fragments sampled for PCR may not have contained parasites, a sampling limitation analogous to amastigote detection in bone-marrow aspirates in visceral leishmaniasis. We also expand the discussion of alternative routes, including intrapartum exposure to parasite-harboring amniotic fluid, maternal blood, or genital-tract secretions, and note that distinguishing true transplacental transmission from peripartum acquisition will require longitudinal mother–infant sampling with quantitative and more extensive placental analysis (Discussion, p. 15, lines 379–393).

# Editor / Journal Requirements

**Data availability (Requirement 5).** We have deposited the dataset underlying this study in the Harvard Dataverse repository under a compatible CC-BY license as per PLoS NTP requirements, and the manuscript now provides the persistent DOI (<https://doi.org/10.7910/DVN/AWNKPA>) in the Data Availability statement. The data have been de-identified so that they can be shared publicly without compromising participant confidentiality. Nonetheless, we are still pending formal approval from our institutional review board to be able to grant unrestricted access. At the moment, access can be requested on a case-by-case basis to the corresponding author through the Harvard Dataverse by creating an account. Should the data be required by the editorial team or the editors, we will be happy to provide access to the dataset upon request. As stated before, the full analysis and manuscript source code is openly available at <https://github.com/javimangal/leishmania-placental>, but it should be noted that minor code adaptations would be needed for the R scripts to run with the new variables which were categorized as part of a data minimization procedure to avoid participant re-identification (e.g., mother’s age and gestational age were categorized), although the Harvard Dataverse contains scripts adapted to reproduce analyses with the minimized data. The geographical analyses cannot be reproduced as sharing participant place of residence data together with other individual participant data would increase the risk of participant re-identification.

**Financial disclosure (Requirements 6 & 7).** We have revised the Financial Disclosure to state author initials alongside each funding source, with grant numbers and funder URLs, and have confirmed the order of funders in the submission form. The revised statement, which now appears in the manuscript (Funding, p. 16–17, lines 449–459), reads: “This work was supported by the Instituto Nacional de Perinatología (<https://www.gob.mx/salud/inper>) under grant #2017-3-116 to NGS; the Indiana University President’s International Research Award (<https://global.iu.edu/>) under grants #PSA-PRO-0680-2020; PIRA10302019 to RXA and MMW; the University of Chicago Pritzker School of Medicine Pritzker Fellowship (<https://pritzker.uchicago.edu/>) to BAB; and the American Society of Tropical Medicine and Hygiene Benjamin H. Kean Travel Fellowship (<https://www.astmh.org/awards-fellowships-medals/astmh-sponsored-fellowships/benjamin-h-kean-travel-fellowship>) to BAB. The funders had no role in study design, data collection and analysis, decision to publish, or preparation of the manuscript.”

**Competing interests (Requirement 8).** We confirm the journal-amended statement is correct, and we have updated the manuscript to read: “The authors have declared that no competing interests exist.” (Declaration of interests, p. 17, line 480).

**Blot/gel original images (Requirement 9).** The original, uncropped agarose gel image underlying Fig 2 is provided with the revision. No cropping or intensity adjustment was applied beyond uniform brightness/contrast across the whole image. These same source data underlie the densitometric quantification requested by Reviewer 1 (point 3). We have also reconciled the control-lane labelling between the figure legend and the original gel.

**Fig 1 — map base layer and legend (Potential Copyright Issue 2).** The map is generated entirely in R from openly licensed sources, now cited directly in the Fig 1 legend (p. 5): Natural Earth 1:10m cultural vectors (public domain) for the national and state outlines, and INEGI open-data layers (municipal boundaries, land use and vegetation, and road network) under Mexico’s open-data licence. Water bodies are shown as the inland-water areas of the same INEGI land use and vegetation layer, so no separate water dataset is used thus removing the dependency on the CONABIO dataset. No proprietary basemap was used. The small caption that had been embedded inside the Fig 1 image file has been removed in the figure-generating script; that information is now presented in the manuscript figure legend at standard text size, which also resolves Reviewer 2’s legibility comment.

**Fig 1 — data-source URLs (Editorial Office point i).** We thank the editorial office for noting that the previously listed INEGI URLs returned error pages. We have replaced every INEGI link in the Fig 1 legend with its English-language equivalent on INEGI’s international site (en.www.inegi.org.mx), retaining the same product identifiers, and we have pointed the licence link to the English “Terms of Free Use of Information” page (<https://en.www.inegi.org.mx/inegi/terminos.html>). We opened each English-language link and confirmed that it is accessible and resolves to the relevant INEGI page.

**Former sampling-schematic figure — clip-art (Potential Copyright Issue 1).** In agreement with Reviewer 2, the sampling-schematic figure has been removed and the sampling procedure is described in the Methods text. This also resolves the clip-art copyright query. All references to it have been removed and the remaining figures renumbered.

# Additional Revisions

**Fuller reporting of the sequencing results.** As described in the Methods (Sequencing), molecular identification of *Leishmania* relied on two independent targets processed in two laboratories: sequencing of the eluted minicircle bands at the Instituto de Biotecnología, Universidad Nacional Autónoma de México (UNAM), and amplification and sequencing of the ITS1/5.8S ribosomal region at the Indiana University-Bloomington Global Environmental Health Laboratory. The original submission reported only the three samples characterized at UNAM. For completeness, the Results now also report the two additional placental samples in which the ITS1 assay identified *L. mexicana* (97–99% homology), which had not been among the three sequenced at UNAM. Because both molecular targets and both laboratories are already described in the Methods, this addition reflects fuller reporting of results generated by the methods already disclosed rather than any new experiment (Results, Confirmation and Identification of *Leishmania* by Sequencing, p. 10).

**Corrected citation (Introduction).** The reference supporting the visceralization of *L. mexicana* in a young child has been corrected to the study by Monroy-Ostria et al. that identified this species as the cause of visceral leishmaniasis in an infant from Tabasco (Monroy-Ostria et al. 2000), and the species attribution has been clarified accordingly.

We believe these revisions have substantially strengthened the manuscript and hope it is now suitable for publication in *PLOS Neglected Tropical Diseases*. We thank the editors and reviewers again for their time and constructive input.

Sincerely,

**Dra. Norma Galindo-Sevilla**, on behalf of all co-authors
norma.galindo@inper.gob.mx
<https://orcid.org/0000-0002-6639-8475>
Departamento de Infectología e Inmunología, Instituto Nacional de Perinatología, Secretaría de Salud. Ciudad de México, Mexico.

# References

Armijos, Rodrigo X., Brandon A. Berger, Alejandra González Ayala, et al. 2026. “Placentas from Healthy Women Living in a Cutaneous Leishmaniasis Endemic Region in Mexico Harbor Leishmania mexicana Parasites: Potential Implications for Pregnancy and Neonatal Outcomes.” *medRxiv*, ahead of print. <https://doi.org/10.64898/2026.05.11.26352970>.

Galindo-Sevilla, Norma del C. 2022. “Bases for a treatment of cutaneous leishmaniasis with moderate hyperthermia.” *Gaceta Medica de Mexico* 158 (4). <https://doi.org/10.24875/gmm.m22000674>.

Monroy-Ostria, Amalia, Omar Hernandez-Montes, and Douglas C Barker. 2000. “Aetiology of Visceral Leishmaniasis in Mexico.” *Acta Tropica* 75 (2): 155–61. <https://doi.org/10.1016/s0001-706x(99)00055-8>.

Torres-Guerrero, Edoardo, Marco Romano Quintanilla-Cedillo, Julieta Ruiz-Esmenjaud, and Roberto Arenas. 2017. “Leishmaniasis: A Review.” *F1000Research* 6 (May): 750. <https://doi.org/10.12688/f1000research.11120.1>.
