# Supplementary material for: Placentas from healthy women living in a cutaneous leishmaniasis endemic region in Mexico harbor Leishmania mexicana parasites: Potential implications for pregnancy and neonatal outcomes
Source: PLoS Negl Trop Dis. 2026 Sep 15;20(9):e0014367. doi: 10.1371/journal.pntd.0014367 (PMC13588509; doi:10.1371/journal.pntd.0014367)
Supplement: Peer review history

# Peer review: Decision Letter 0

**DOI:** 10.1371/journal.pntd.0014367.r001

**Authors:** Andrés F. Henao-Martínez, Susan Madison-Antenucci

18 Jun 2026

PNTD-D-26-00898

Placentas from healthy women living in a cutaneous leishmaniasis endemic region in Mexico harbor Leishmania mexicana parasites: Potential implications for pregnancy and neonatal outcomes

PLOS Neglected Tropical Diseases

Dear Dr. Galindo-Sevilla,

Thank you for submitting your manuscript to PLOS Neglected Tropical Diseases. After careful consideration, we feel that it has merit but does not fully meet PLOS Neglected Tropical Diseases's publication criteria as it currently stands. Therefore, we invite you to submit a revised version of the manuscript that addresses the points raised during the review process.

Please submit your revised manuscript within by Aug 16 2026 11:59PM. If you will need more time than this to complete your revisions, please reply to this message or contact the journal office at plosntds@plos.org. When you're ready to submit your revision, log on to https://www.editorialmanager.com/pntd/ and select the 'Submissions Needing Revision' folder to locate your manuscript file.

Please include the following items when submitting your revised manuscript:

* A letter that responds to each point raised by the editor and reviewer(s). You should upload this letter as a separate file labeled 'Response to Reviewers'. This file does not need to include responses to any formatting updates and technical items listed in the 'Journal Requirements' section below.

* A marked-up copy of your manuscript that highlights changes made to the original version. You should upload this as a separate file labeled 'Revised Manuscript with Track Changes'.

* An unmarked version of your revised paper without tracked changes. You should upload this as a separate file labeled 'Manuscript'.

If you would like to make changes to your financial disclosure, competing interests statement, or data availability statement, please make these updates within the submission form at the time of resubmission. Guidelines for resubmitting your figure files are available below the reviewer comments at the end of this letter.

As the corresponding author, your ORCID iD is verified in the submission system and will appear in the published article. PLOS supports the use of ORCID, and we encourage all coauthors to register for an ORCID iD and use it as well. Please encourage your coauthors to verify their ORCID iD within the submission system before final acceptance, as unverified ORCID iDs will not appear in the published article. Only the individual author can complete the verification step; PLOS staff cannot verify ORCID iDs on behalf of authors.

We look forward to receiving your revised manuscript.

Kind regards,

Andrés F. Henao-Martínez, M.D.

Academic Editor

PLOS Neglected Tropical Diseases

Susan Madison-Antenucci

Section Editor

PLOS Neglected Tropical Diseases

Shaden Kamhawi

co-Editor-in-Chief

PLOS Neglected Tropical Diseases

orcid.org/0000-0003-4304-636XX

Paul Brindley

co-Editor-in-Chief

PLOS Neglected Tropical Diseases

orcid.org/0000-0003-1765-0002

**Additional Editor Comments (if provided)**:

**Journal Requirements**:

If the reviewer comments include a recommendation to cite specific previously published works, please review and evaluate these publications to determine whether they are relevant and should be cited. There is no requirement to cite these works unless the editor has indicated otherwise.

1) We ask that a manuscript source file is provided at Revision. Please upload your manuscript file as a .doc, .docx, .rtf or .tex. If you are providing a .tex file, please upload it under the item type u2018LaTeX Source Fileu2019 and leave your .pdf version as the item type u2018Manuscriptu2019.

2) We do not publish any copyright or trademark symbols that usually accompany proprietary names, eg ©,  ®, or TM  (e.g. next to drug or reagent names). Therefore please remove all instances of trademark/copyright symbols throughout the text, including:

- ® on page: 7

- TM on page: 7.

3) Please upload all main figures as separate Figure files in .tif or .eps format. For more information about how to convert and format your figure files please see our guidelines:

https://journals.plos.org/plosntds/s/figures

4) Please upload a copy of Figure Fig 1-5 which you refer to in your text on pages 4, 6, 7, and 9 and 12. Or, if the figure is no longer to be included as part of the submission please remove all reference to it within the text.

5) In the online submission form, you indicated that The data underlying this study contain sensitive patient information and cannot be shared publicly to protect participant confidentiality. Deidentified data may be made available upon reasonable request to the corresponding author (norma.galindo@inper.gob.mx).. All PLOS journals now require all data underlying the findings described in their manuscript to be freely available to other researchers, either

1. In a public repository

2. Within the manuscript itself

3. Uploaded as supplementary information.

This policy applies to all data except where public deposition would breach compliance with the protocol approved by your research ethics board. If your data cannot be made publicly available for ethical or legal reasons (e.g., public availability would compromise patient privacy), please explain your reasons by return email and your exemption request will be escalated to the editor for approval. Your exemption request will be handled independently and will not hold up the peer review process, but will need to be resolved should your manuscript be accepted for publication. One of the Editorial team will then be in touch if there are any issues.

6) Please amend your detailed Financial Disclosure statement. This is published with the article. It must therefore be completed in full sentences and contain the exact wording you wish to be published.

i) State the initials, alongside each funding source, of each author to receive each grant. For example: "This work was supported by the National Institutes of Health (####### to AM; ###### to CJ) and the National Science Foundation (###### to AM).".

If you did not receive any funding for this study, please simply state: u201cThe authors received no specific funding for this work.u201d

7) Your current Financial Disclosure states, “This study was supported by funding from the National Perinatology Institute to Dr. Galindo-Sevilla (grant #2017-3-116) and from the Indiana University President's International Research Award to Drs. Armijos and Weigel (grant #PSA-PRO-0680-2020; PIRA10302019). Dr. Berger was supported by the University of Chicago Pritzker School of Medicine Pritzker Fellowship and the American Society of Tropical Medicine and Hygiene Benjamin H. Kean Travel Fellowship. The institutional funders had no role in the study design, data collection, analysis, decision to publish, or preparation of the manuscript.” However, in your funding information on the submission form, "order of grants" mismatches. Please indicate by return email the full and correct funding information for your study and confirm the order in which funding contributions should appear. Please be sure to indicate whether the funders played any role in the study design, data collection and analysis, decision to publish, or preparation of the manuscript.

8) We have amended your Competing Interest statement to comply with journal style. We kindly ask that you double check the statement and let us know if anything is incorrect.

9) PLOS now requires that authors provide the original uncropped and unadjusted images underlying all blot or gel results reported in a submission’s figures or Supporting Information files. This policy and the journal’s other requirements for blot/gel reporting and figure preparation are described in detail at https://journals.plos.org/plosone/s/figures#loc-blot-and-gel-reporting-requirements and https://journals.plos.org/plosone/s/figures#loc-preparing-figures-from-image-files.

When you submit your revised manuscript, please ensure that your figures adhere fully to these guidelines and provide the original underlying images for all blot or gel data reported in your submission. See the following link for instructions on providing the original image data: https://journals.plos.org/plosone/s/figures#loc-original-images-for-blots-and-gels.

In your cover letter, please note whether your blot/gel image data are in Supporting Information or posted at a public data repository, provide the repository URL if relevant, and provide specific details as to which raw blot/gel images, if any, are not available. Email us at customercare@plos.org if you have any questions.

**Reviewers' Comments**:

Reviewer's Responses to Questions

**Key Review Criteria Required for Acceptance**?

As you describe the new analyses required for acceptance, please consider the following:

**Methods**

-Are the objectives of the study clearly articulated with a clear testable hypothesis stated?

-Is the study design appropriate to address the stated objectives?

-Is the population clearly described and appropriate for the hypothesis being tested?

-Is the sample size sufficient to ensure adequate power to address the hypothesis being tested?

-Were correct statistical analysis used to support conclusions?

-Are there concerns about ethical or regulatory requirements being met?

Reviewer #1: The methods performed are not adequate and therefore more methods and anlysis has been suggested

Reviewer #2: -Are the objectives of the study clearly articulated with a clear testable hypothesis stated? YES

-Is the study design appropriate to address the stated objectives? YES

-Is the population clearly described and appropriate for the hypothesis being tested? YES

-Is the sample size sufficient to ensure adequate power to address the hypothesis being tested? YES

-Were correct statistical analysis used to support conclusions? YES

-Are there concerns about ethical or regulatory requirements being met? NO

**********

**Results**

-Does the analysis presented match the analysis plan?

-Are the results clearly and completely presented?

-Are the figures (Tables, Images) of sufficient quality for clarity?

Reviewer #1: Results are suggested to be revised and must be reperfrmed

Reviewer #2: -Does the analysis presented match the analysis plan? YES

-Are the results clearly and completely presented? YES

-Are the figures (Tables, Images) of sufficient quality for clarity? YES

**********

**Conclusions**

-Are the conclusions supported by the data presented?

-Are the limitations of analysis clearly described?

-Do the authors discuss how these data can be helpful to advance our understanding of the topic under study?

-Is public health relevance addressed?

Reviewer #1: Somewhat matches but should be revised as per the comments

Reviewer #2: -Are the conclusions supported by the data presented? YES

-Are the limitations of analysis clearly described? YES, see reviewer comments bellow

-Do the authors discuss how these data can be helpful to advance our understanding of the topic under study? YES

-Is public health relevance addressed? YES

**********

**Editorial and Data Presentation Modifications?**

Use this section for editorial suggestions as well as relatively minor modifications of existing data that would enhance clarity. If the only modifications needed are minor and/or editorial, you may wish to recommend “Minor Revision” or “Accept”.

Reviewer #1: (No Response)

Reviewer #2: The font size in the caption for Figure 1 is too small.

**********

**Summary and General Comments**

Use this section to provide overall comments, discuss strengths/weaknesses of the study, novelty, significance, general execution and scholarship. You may also include additional comments for the author, including concerns about dual publication, research ethics, or publication ethics. If requesting major revision, please articulate the new experiments that are needed.

Reviewer #1: Authors are suggested to work on the following comments and resubmit the manuscript. Manuscripts lacks some clarity and also proper result representation; quantification are missing.

1.Authors are suggested to add treatment options for leishmaniasis which is missing in the introduction section, recent studies have suggested potential gene KO strategies for vaccine candidates and various plant-based therapies are in use for decades. Authors are suggested to cite these publications: PMID: 40196396, PMID: 33528770, https://link.springer.com/article/10.1007/s11756-024-01656-y

2. The authors have included women with a singleton fetus pregnancy. Please provide a rationale for the same. Also the positive cases have any history of prior fetus with positive Leishmaniasis? If not, what could have been the cause of the congenital transmission this time?

3. Figure 3, appears to be a representative image from one patient sample collected across the placenta and other controls run on agarose gel. It is suggested to quantify the patient samples’ Agarose gel bands with respect (densitometric analysis), to the positive control lane 17, and possible quantification should be shown across other samples.

Also how do the light and dark bands of 120bp gene in patient samples represent the disease outcome and how does that correlate with histopathology or other data? This must be discussed in the results and discussion.

4. Figure 4, A and B image legends do not match the image description. What does image 4B represent if it’s the same image, show the arrow with macrophage and intracellular parasite. Scale bar is required in the images. The parasite inside the macrophage should be quantified or the infected macrophages corresponding to Figure 4. Hand E stain of the placenta should also be incorporated.

Reviewer #2: The subject of this study is extremely relevant to public health, especially in countries endemic for leishmaniasis, and is still under-researched. A few suggestions are presented below.

Introduction

Page 3, line 47:

“Protozoan infections (malaria, toxoplasmosis, Chagas’ disease, and visceral 47

leishmaniasis)”. I suggest changing to “Diseases caused by protozoa (malaria, toxoplasmosis, Chagas, and visceral leishmaniasis)” or write the scientific names of the protozoa (not the disease) in parentheses.

MATERIAL AND METHODS

Page 4, line 71:

The font size in the caption for Figure 1 is too small.

Page 6, Figure 2:

In my opinion, this figure is unnecessary; the methodology for collecting samples from the placenta and buccal mucosa of newborns is clear and already described in the paragraph (lines 123-135).

RESULTS

Page 9, line 209:

Most (37 of 53; 96%) of the study participants….; 37 out of 53 participants is 68.9%

Page 9, lines 218-221:

“In five patients, both the internal and external placental regions were positive; the internal region tested positive in 13 samples (8 showing intense bands, 5 faint bands), while the external

region tested positive in 10 samples (5 intense, 5 faint; Figure 3).”

Figure 3 is a representative gel sample from the PCR analysis, as it shows the results of some placental samples and not all 18 positive samples. I suggest also changing the text to/or something similar to; “

“PCR positivity was observed for both the internal and external placental regions in 5 samples, exclusively for the internal region in …. samples, and solely for the external region in …. samples (Figure 3)”

DISCUSSION

Page 14, lines 317-320:

“The detection of parasites by PCR does not distinguish between live and dead

parasites, and this study did not include parasite cultures from placental samples.”

Are there histological sections stained with hematoxylin-eosin? It would be interesting to show if there was inflammatory cellular infiltrate around the parasitized cells. It would also be interesting to macerate part of the infected tissues and inoculate them into culture medium or even infect laboratory animals to assess the viability/infectivity of amastigotes revealed by immunofluorescence. This could indicate that the congenital infection occurs from amastigotes present in the placenta, supporting the plausibility of hematogenous transmission to the newborn’s oral mucosa.

Page 14, lines 332-336:

“Notably, 8 of the 11 buccal PCR-positive newborns were born from PCR-negative placenta mothers, suggesting that the placenta is not the only potential route and that contamination at the time of delivery cannot be excluded. A longitudinal follow-up of this cohort could provide valuable insights into potential long-term effects on the offspring’s immune tolerance”

Isn't it more likely that the parasite load was low or the distribution of amastigotes in the placenta was irregular, and the tissue sample collected for PCR did not contain amastigotes, even though the pregnant woman's placenta was infected?

In bone marrow aspirations from patients with visceral leishmaniasis, these aspects can lead to variable results in the identification of amastigotes.

What other infection mechanisms could occur, including those during childbirth? Authors have mentioned it. Discuss this further.

**********

PLOS authors have the option to publish the peer review history of their article (what does this mean?). If published, this will include your full peer review and any attached files.

If you choose “no”, your identity will remain anonymous but your review may still be made public.

**Do you want your identity to be public for this peer review?**  For information about this choice, including consent withdrawal, please see our Privacy Policy.

Reviewer #1: **Yes:**  NAMRATA ANAND

Reviewer #2: No

[NOTE: If reviewer comments were submitted as an attachment file, they will be attached to this email and accessible via the submission site. Please log into your account, locate the manuscript record, and check for the action link "View Attachments". If this link does not appear, there are no attachment files.]

**Figure resubmission**:

**Reproducibility**:

To enhance the reproducibility of your results, we recommend that authors of applicable studies deposit laboratory protocols in protocols.io, where a protocol can be assigned its own identifier (DOI) such that it can be cited independently in the future. Additionally, PLOS ONE offers an option to publish peer-reviewed clinical study protocols. Read more information on sharing protocols at https://plos.org/protocols?utm_medium=editorial-email&utm_source=authorletters&utm_campaign=protocols

# Author comment: Author response to Decision Letter 1

**DOI:** 10.1371/journal.pntd.0014367.r002

29 Jul 2026

Attachment Submitted filename: response_to_reviewers.docx

# Editor report: Decision Letter 1

**DOI:** 10.1371/journal.pntd.0014367.r003

**Authors:** Andrés F. Henao-Martínez, Susan Madison-Antenucci

31 Aug 2026

Dear Dr. Galindo-Sevilla,

We are pleased to inform you that your manuscript 'Placentas from healthy women living in a cutaneous leishmaniasis endemic region in Mexico harbor Leishmania mexicana parasites: Potential implications for pregnancy and neonatal outcomes' has been provisionally accepted for publication in PLOS Neglected Tropical Diseases.

Before your manuscript can be formally accepted you will need to complete some formatting changes, which you will receive in a follow up email. A member of our team will be in touch with a set of requests.

Please note that your manuscript will not be scheduled for publication until you have made the required changes, so a swift response is appreciated.

IMPORTANT: The editorial review process is now complete. PLOS will only permit corrections to spelling, formatting or significant scientific errors from this point onwards. Requests for major changes, or any which affect the scientific understanding of your work, will cause delays to the publication date of your manuscript.

Should you, your institution's press office or the journal office choose to press release your paper, you will automatically be opted out of early publication. We ask that you notify us now if you or your institution is planning to press release the article. All press must be co-ordinated with PLOS.

Thank you again for supporting Open Access publishing; we are looking forward to publishing your work in PLOS Neglected Tropical Diseases.

Best regards,

Andrés F. Henao-Martínez, M.D

Academic Editor

PLOS Neglected Tropical Diseases

Susan Madison-Antenucci

Section Editor

PLOS Neglected Tropical Diseases

Shaden Kamhawi

co-Editor-in-Chief

PLOS Neglected Tropical Diseases

orcid.org/0000-0003-4304-636XX

Paul Brindley

co-Editor-in-Chief

PLOS Neglected Tropical Diseases

orcid.org/0000-0003-1765-0002

***********************************************************

# Editor report: Acceptance letter

**DOI:** 10.1371/journal.pntd.0014367.r004

**Authors:** Andrés F. Henao-Martínez, Susan Madison-Antenucci

Dear Dr. Galindo-Sevilla,

We are delighted to inform you that your manuscript, "Placentas from healthy women living in a cutaneous leishmaniasis endemic region in Mexico harbor Leishmania mexicana parasites: Potential implications for pregnancy and neonatal outcomes," has been formally accepted for publication in PLOS Neglected Tropical Diseases.

We have now passed your article onto the PLOS Production Department who will complete the rest of the publication process. All authors will receive a confirmation email upon publication.

The corresponding author will soon be receiving a typeset proof for review, to ensure errors have not been introduced during production. Please review the PDF proof of your manuscript carefully, as this is the last chance to correct any scientific or type-setting errors. Please note that major changes, or those which affect the scientific understanding of the work, will likely cause delays to the publication date of your manuscript. Note: Proofs for Front Matter articles (Editorial, Viewpoint, Symposium, Review, etc...) are generated on a different schedule and may not be made available as quickly.

Soon after your final files are uploaded, the early version of your manuscript will be published online unless you opted out of this process. The date of the early version will be your article's publication date. The final article will be published to the same URL, and all versions of the paper will be accessible to readers.

For Research Articles, you will receive an invoice from PLOS for your publication fee after your manuscript has reached the completed accept phase. If you receive an email requesting payment before acceptance or for any other service, this may be a phishing scheme. Learn how to identify phishing emails and protect your accounts at https://explore.plos.org/phishing.

Thank you again for supporting open-access publishing; we are looking forward to publishing your work in PLOS Neglected Tropical Diseases.

Best regards,

Shaden Kamhawi

co-Editor-in-Chief

PLOS Neglected Tropical Diseases

Paul Brindley

co-Editor-in-Chief

PLOS Neglected Tropical Diseases
